# Tinkering Evolution of Post-Transcriptional RNA Regulons: Puf3p in Fungi as an Example

**DOI:** 10.1371/journal.pgen.1001030

**Published:** 2010-07-22

**Authors:** Huifeng Jiang, Wenjun Guan, Zhenglong Gu

**Affiliations:** 1 Division of Nutritional Sciences, Cornell University, Ithaca, New York, United States of America; 2 College of Life Sciences, Zhejiang University, Hangzhou, China; University of Michigan, United States of America

## Abstract

Genome-wide studies of post-transcriptional mRNA regulation in model organisms indicate a “post-transcriptional RNA regulon” model, in which a set of functionally related genes is regulated by mRNA–binding RNAs or proteins. One well-studied post-transcriptional regulon by Puf3p functions in mitochondrial biogenesis in budding yeast. The evolution of the Puf3p regulon remains unclear because previous studies have shown functional divergence of Puf3p regulon targets among yeast, fruit fly, and humans. By analyzing evolutionary patterns of Puf3p and its targeted genes in forty-two sequenced fungi, we demonstrated that, although the Puf3p regulon is conserved among all of the studied fungi, the dedicated regulation of mitochondrial biogenesis by Puf3p emerged only in the *Saccharomycotina* clade. Moreover, the evolution of the Puf3p regulon was coupled with evolution of codon usage bias in down-regulating expression of genes that function in mitochondria in yeast species after genome duplication. Our results provide a scenario for how evolution like a tinker exploits pre-existing materials of a conserved post-transcriptional regulon to regulate gene expression for novel functional roles.

## Author Summary

It is well known that the evolution of gene expression can account for significant phenotypic diversity among species. Gene expression is regulated at various levels. Many studies have demonstrated gene expression changes caused by mutations in transcription. Post-transcriptional regulation is also crucial for splicing, translation, localization, and degeneration of mRNAs in eukaryotes and, thus, is important in determining the abundance of gene expression. Changes in post-transcriptional regulons are also important in the evolution of gene expression. In this study we investigated the evolution of a particular post-transcriptional regulon, Puf3p, in fungal species. Our results illustrated an important evolutionary mode for evolution of post-transcriptional regulon, *i.e.*, pre-existing materials of conserved post-transcriptional regulons can be recruited to regulate gene expression for novel functional roles.

## Introduction

Evolution of gene expression may account for significant phenotypic diversity among species [1]–[6]. Gene expression is regulated at various levels. Many studies have demonstrated gene expression changes caused by mutations in transcription [7], [8]. Post-transcriptional regulation is also crucial for splicing, translation, localization and degeneration of mRNAs in eukaryotes, and thus is important in determining the abundance of gene expression [9], [10]. It is likely that changes in post-transcriptional regulons are also important in the evolution of gene expression [11].

The control of post-transcriptional regulation is mediated by regulons such as mRNA-binding proteins (RBP) or RNAs (e.g., microRNAs) which usually bind to elements in the 3′ untranslated regions (UTR) and determine the fate of their targeted mRNAs. Evolution of microRNA post-transcriptional regulons has been well studied due to recent improvement in understanding their functions. It was shown that novel microRNAs can turn over rapidly during evolution [12], and for those that are highly conserved over long evolutionary distances, their targets can change dramatically even within populations [13]. Studies on microRNAs have revealed interesting information on evolution of this particular type of post-transcriptional regulon, whereas evolution of RBP regulons remains poorly understood. Furthermore, RBP regulons play major post-transcriptional roles in budding yeast because the species lost the microRNA regulatory machine [14].

One of the best-characterized RBP families is PUF (Pumilio and FBF, FBF represents for *fem*-3 binding factor), which is conserved in a wide variety of eukaryotes from yeast to humans [15]–[19]. The PUF post-transcriptional regulon regulates diverse gene sets in various model organisms. For example, in the budding yeast, *Saccharomyces cerevisiae*, genes most commonly targeted by Puf3p are in the mitochondria and play essential roles in mitochondrial biogenesis [17], [20]. In the fruit fly, *Drosophila melanogaster*, Pumilio (a PUF protein), which binds with the same element as Puf3p in the budding yeast, is necessary for early embryogenesis and development of primordial germ cells [21], . Genome-wide identification of the Pumilio targets in fruit flies uncovered genes involved particularly in nucleotide metabolism, transcriptional regulation and synthesis of membrane proteins [18]. In humans, two paralogous PUF proteins (Pum1p and Pum2p), which interact with the microRNA system in post-transcriptional regulation, share the same binding-element with yeast Puf3p and bind to mRNAs from genes that function in transcriptional regulation and cell proliferation [19], [23].

Previous studies have reported that the binding site of Puf3p is highly conserved in *sensu stricto* yeasts [24]–[26]. Taking advantage of a large number of genomic sequences, in this study we investigated the evolution of the Puf3p post-transcriptional regulon in fungi. Our results show continuous steps of functional innovation in the Puf3p regulon despite its ultra conservation in these fungal species. First, the regulation of mitochondrial biogenesis by the Puf3p regulon originated in the *Saccharomycotina* subdivision; second, the Puf3p regulon was coupled with codon usage bias to modulate expression of genes that function in mitochondria in yeasts after whole genome duplication (WGD). Our work and reports from other labs show that mitochondria underwent significant functional changes during the origin of an efficient aerobic fermentation system in the yeast species that went through WGD [27]–[30]. This current report provides evidence suggesting that the Puf3p post-transcriptional regulon was involved in the evolution of this novel life history in yeasts.

## Results

### The binding element of the Puf3p regulon is conserved in fungi

**Figure 1:**
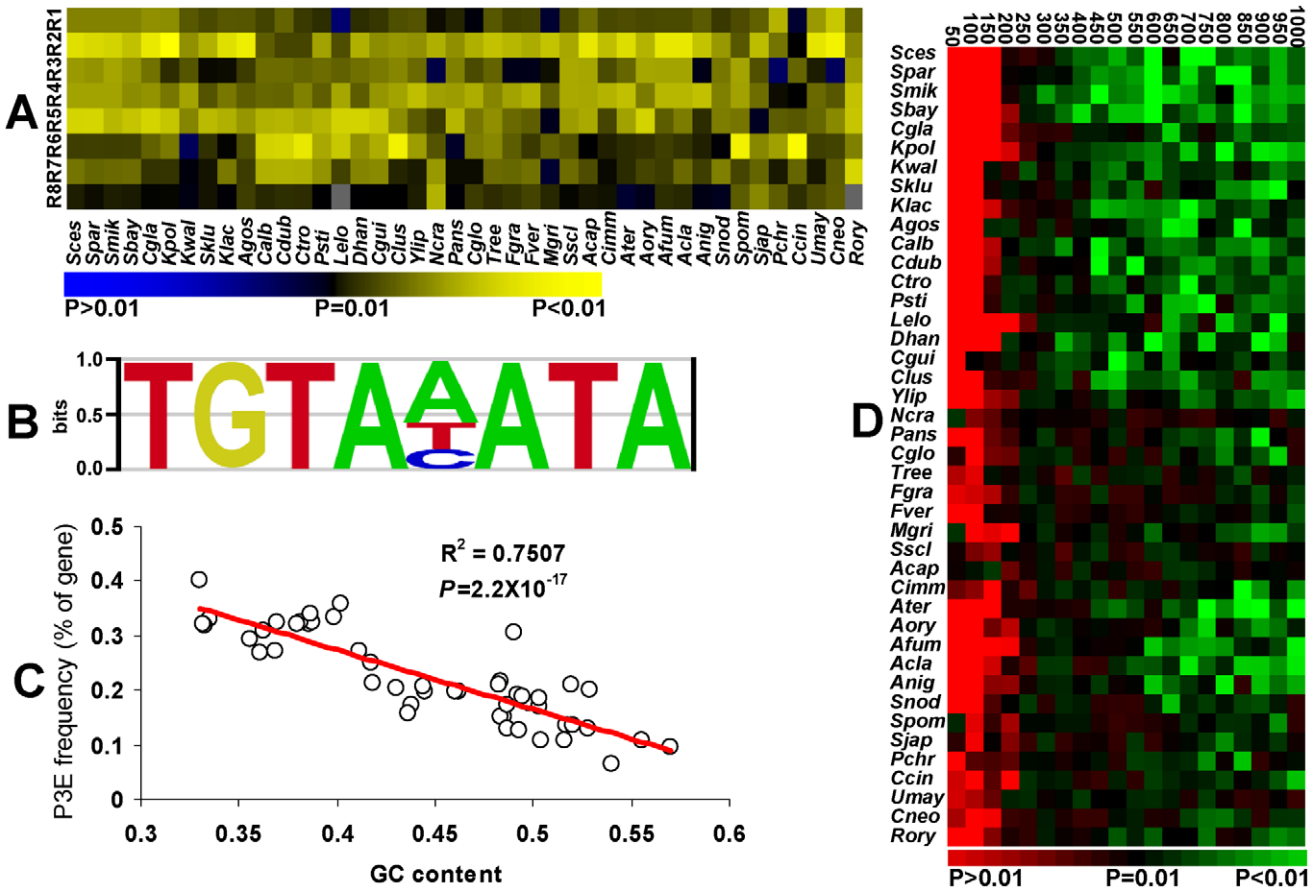
Conservation of the Puf3p regulon in fungi. (A) Conservation of the pumilio domain. R1–R8 represents the repeat domains. Different colors indicate the *P* values of domain identification by SMART alignment. The two missing domains were depicted in grey. The full name for each species was listed in Table S1. (B) The motif of P3E in the budding yeast. 3′ UTR from mitochondrial ribosomal proteins were used to reconstruct the P3E profile by MEME [58]. The multiple sequence alignment of P3E from the MEME output was used to get the P3E logo in enoLOGOS [59]. (C) Genome-wide GC content and the P3E frequency. Linear regression line was shown on the figure with R^2^ and *P* values. (D) The occurrences of the P3E at the downstream of all annotated genes in each species. Different colors show the *P* values of having the observed frequencies in each sliding window of studied species.

The RNA-binding domain of Puf3p, called the PUF homology domain (PUF-HD), consists of eight repeated peptide motifs [31]–[33]. In order to study the evolution of the RNA-binding domain, orthologous genes of PUF3 were identified from the forty-two sequenced fungal species (Table S1). Domain alignments in SMART [34], [35] indicate that almost all Puf3p orthologs contain the eight repeated motifs even though some repeats are not highly conserved (Figure 1A). Puf3p in *Lodderomyces elongisporus* and *Rhizopus oryzae* lost one repeat which may have resulted from insufficient genome sequencing or assembly because both orthologs are located at the end of the assembled contigs. This result demonstrates that the binding domain of Puf3p is conserved among all studied fungi.

The evolutionary trajectory of the Puf3p-binding element in its target genes was further investigated. Because the 8nt-core motif of P3E is conserved from yeast to human, we used the 8nt-core P3E profiling in the budding yeast as a reference to identify all possible puf3p targeted genes that contain at least one P3E at their 3′ downstream sequences (Figure 1B) [19]. Because GC content is very low in the P3E, the genomic GC content would inevitably affect the frequency of P3E in each species. Indeed, as shown in Figure 1C, at the genome level, the number of genes with P3E is negatively correlated with the genomic GC content among the studied species.

In order to exclude the impact of genomic GC content on our results, we generated 10,000 random sequences for each species based on the average GC content of 1,000 bp downstream sequences of all the annotated genes in this species. Using the occurrence of P3E in these random sequences as background in each species, we estimated the probability of having the observed P3E frequency in the 3′ downstream sequences of all the annotated genes in the same species. The probability was calculated in each sliding window of 50 bp in the 3′ downstream sequences of each species. As shown in Figure 1D, all studied species exhibited significant enrichment of P3E in the first several sliding windows in the 3′ downstream regions. As the 3′ UTR in yeast is usually shorter than 250 bp [36], our results indicate that the enrichment of P3E in the studied fungi results from P3E conservation in the 3′ UTR sequences. When we used the GC contents in the 250 bp regions after the stop codon or in each 50 bp sliding window to calculate the background P3E motif distribution, similar enrichment of P3E motif in the 3′UTR regions are still observed in most fungi species (Figure S1).

### The Puf3p target genes are significantly enriched for mitochondrial functions only in the *Saccharomycotina* subdivision

**Figure 2:**
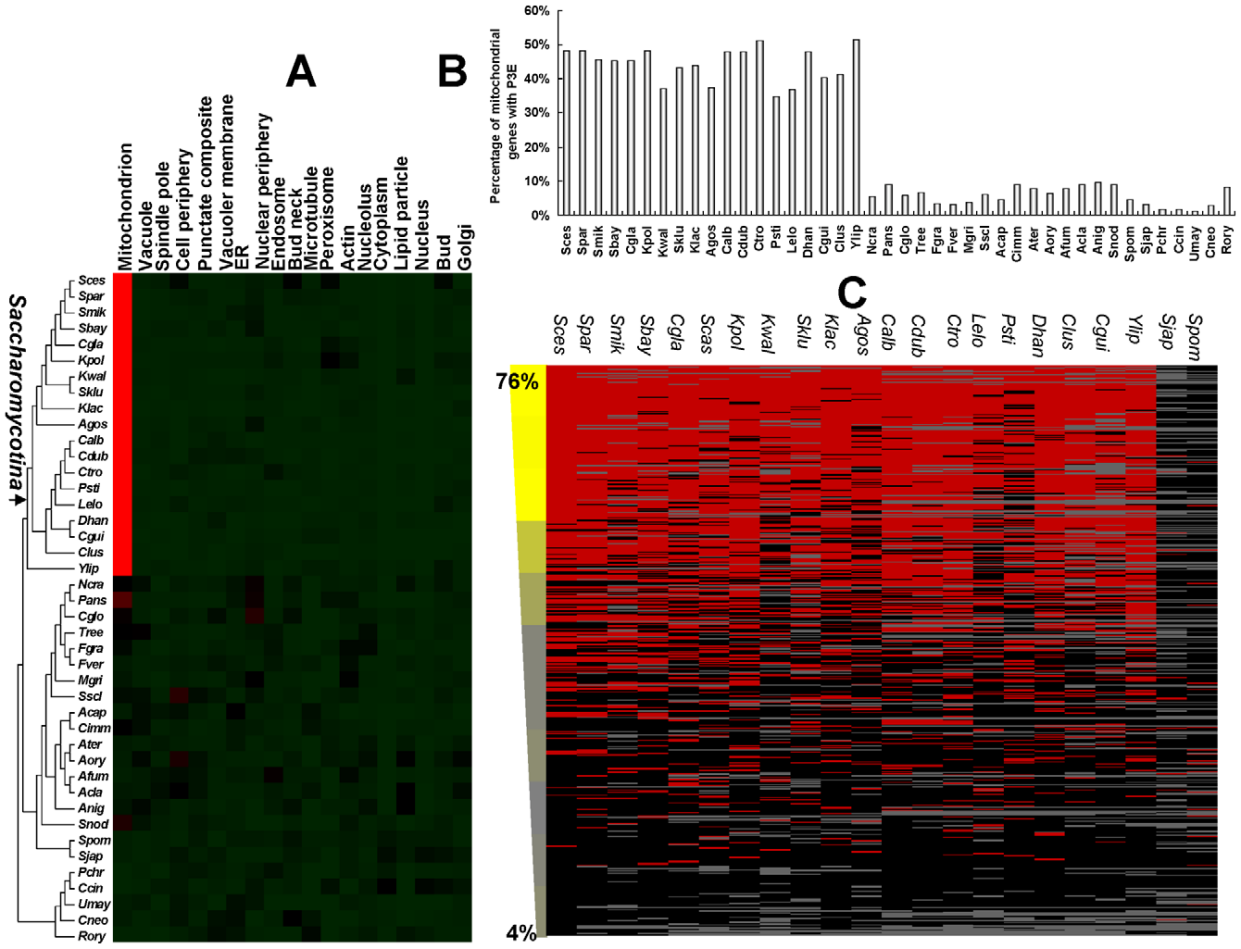
The evolution of the puf3p regulon and mitochondrial biogenesis. (A) Enrichment of P3E in different categories in yeast species. Red color denotes significant enrichment of P3E after multiple test correction (*P*-value<0.01). Black and green denote *P*-value = 0.01 and *P*-value>0.01 respectively. (B) The occurrence of P3E for genes functioning in mitochondria in yeast species. The y axis represents the percentage of P3E conservation in mitochondrial genes for each studied species. (C) Conservation of P3E for genes involved in mitochondrial translation. Each row represents a mitochondrial gene and each column represents a species in the clade *Saccharomycotina* (*S. pombe* and its sister species, *S. japonicus*, which do not have P3E enrichment in mitochondrial genes, were also shown for comparison). The presence and absence of P3E in the downstream of a mitochondrial gene is shown in red and black, respectively. The grey denotes the absence of the corresponding orthologous gene in that species. Genes were sorted by the percentage of having P3E among all orthologs for each gene in the *Saccharomycotina* species (decreasing from top to bottom). The percentages of genes that are involved in mitochondrial translation, which were defined based on the Gene Ontology annotation (GO: 0006412), were calculated in a 50 gene sliding window from top to bottom, and the values were listed at the left of the figure.

Previous reports showed that Puf3p plays an essential role in mitochondrial biogenesis in *S. cerevisiae* [17], [37], [38]. This observation prompted us to investigate whether the functional profile of the Puf3p regulon is also conserved among fungi species. Accordingly, we identified all of orthologous genes between each studied fungal species and budding yeast. Genes in each species are classified into categories based on the sub-cellular localization of their orthologs in the budding yeast [39]. We then estimated the enrichment of genes with P3E in each localization category. As shown in Figure 2A, we discovered that all of the studied species in the *Saccharomycotina* subdivision show significant enrichment of P3E in genes that function in mitochondria. Other clades of the studied fungi did not have this pattern. Indeed, close to 50% of genes that function in mitochondria have P3E in the *Saccharomycotina* subdivision, which is significantly higher than that of species in other clades (Figure 2B, *P*-value = 6×10^−22^). A similar pattern was observed when a slightly different P3E motif profile [17] was used to calculate the motif frequency (Figure S2).

Because regulation of the mitochondrial translational machine is essential for mitochondrial biogenesis [40], we further investigated conservation of P3E among the orthologous genes with this particular function in the *Saccharomycotina* subdivision. Our results showed that ∼80% of genes with highly conserved P3E were involved in mitochondrial translation, whereas this number is only ∼4% for genes with little P3E conservation, indicating that the Puf3p regulation of genes that are involved in the mitochondrial translational machine is highly conserved in these *Saccharomycotina* species (Figure 2C).

### Relaxation of codon usages in Puf3p-regulated mitochondrial genes

**Figure 3:**
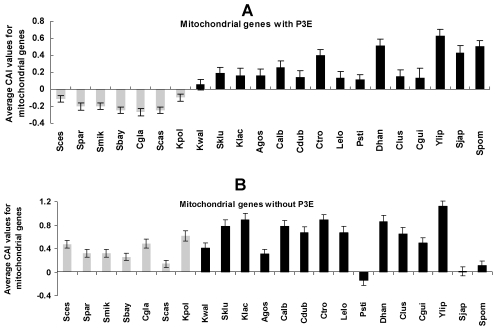
The codon adaptation index (CAI) of mitochondrial genes in species in the clade *Saccharomycotina*. The grey and black bars denote the post and pre-WGD fungi species, respectively. The average CAI values of the mitochondrial genes with P3E (A) and without P3E (B) were shown. The student t-test was used to compare CAI values between pre- and post-WGD fungi species.

Gene expression is regulated at multiple levels. Biased usage of preferred codons can result in enhanced accuracy and speed of protein synthesis in highly expressed genes [41], [42]. Previous studies reported that codon usage bias in mitochondrial genes is relaxed, possibly due to a relaxed function of the organelle with the origin of an efficient aerobic fermentation system in the fungal lineage with WGD [27], [28]. We predicted that Puf3p-regulated mitochondrial genes, due to their importance in mitochondrial biogenesis and functions, would experience more relaxation of codon usage bias than other mitochondrial genes in the post-WGD yeast species. In order to test this, we calculated the average codon bias adaptation index (CAI) for the mitochondrial genes, with and without P3E, for each species. As shown in Figure 3A, the mitochondrial genes with P3E in the post-WGD species show significantly smaller CAI than those genes in the fungal species that diverged from the common ancestor before the WGD event (student t-test, *P* = 3×10^−8^), whereas mitochondrial genes without P3E did not show such a pattern (Figure 3B, student t-test, *P* = 0.1).

### Puf3p regulon in mitochondrial gene regulation

**Figure 4:**
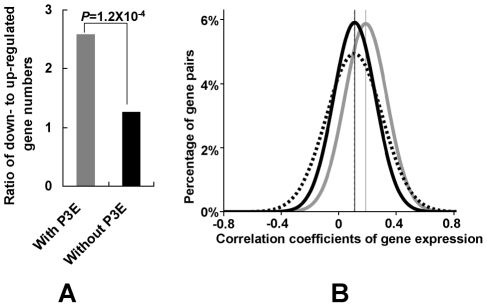
Regulation of mitochondrial genes by Puf3p regulon. (A) Expression change of mitochondrial genes in fermentative conditions. The ratio for the number of down- to up-regulated (1.5 fold difference) gene from the reference to the fermentative condition was shown. *P*-value was obtained by the Fisher's exact test where the numbers of down- and up-regulated genes with P3E were compared to those genes without P3E. (B) The distribution for the pair-wise correlation coefficients of gene expression. The grey line denotes the distribution for the mitochondrial genes with P3E. The dashed line denotes the distribution for the mitochondrial genes without P3E. The black line denotes the distribution between mitochondrial gene pairs one of which has P3E and the other does not have P3E. The student t-test was used to compare different distributions.

Due to the importance of Puf3p regulation in mitochondrial gene degradation, we further investigated expression of its target mitochondrial genes under the fermentative condition. Using gene-expression profiling measured by microarray data [43], we discovered that significantly more mitochondrial genes with P3E were down-regulated in the fermentative medium (YPD) than those without P3E (Figure 4A, Fisher's exact test, *P* = 1.2×10^−4^). Furthermore, we found that mitochondrial genes with P3E tend to be co-regulated because the average correlation coefficients of gene expression among mitochondrial genes with P3E in different conditions is significantly higher than that of genes without P3E (Figure 4B, student t test, *P* = 0). Therefore Puf3p regulon plays an important role in regulating mitochondrial genes in different conditions.

## Discussion

### Function of the Puf3p regulon under various environmental conditions

**Figure 5:**
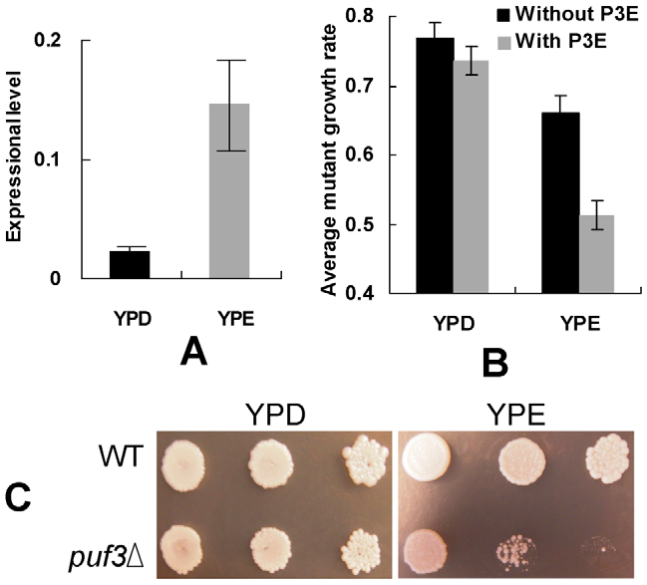
Function of the Puf3p regulon under different conditions. (A) The expression level of PUF3 gene in the fermentative (YPD) and respiratory (YPE) growth conditions. The ACT1 gene was used as a control in the real-time PCR experiments. (B) The average growth rate of gene deletion mutants for the mitochondrial genes with and without P3E in the two conditions. The student t-test was used to compare gene expression (in A) and average growth rate of gene deletion mutants (in B). (C) The growth of *PUF3* gene deletion mutant and the wide-type (WT) strains in the two conditions.

PUF protein was first characterized in *Drosophila* as an mRNA-binding factor that recruits other proteins to inhibit the translation of the bound mRNA [21]. Subsequently, many studies in yeast revealed that the PUF family regulates specific mRNA degeneration by their RNA-binding domains [17], [31], [44], [45]. It was shown that the function of targeting mRNA for degeneration by Puf3p is much more efficient in 2% glucose (YPD, fermentative) than in 3% ethanol (YPE, non-fermentative) [17], [46]. Furthermore, it was shown that Puf3p is crucial for mitochondrial biogenesis and motility under non-fermentative conditions in budding yeast [43]. Saint-Georges and his colleagues reported that Puf3p can transfer its target mRNAs to the peripheral mitochondria in the non-fermentative growth medium [37]. The expression of PUF3 gene is significantly higher in yeast growing in YPE than in YPD (Figure 5A, *P*-value<0.05). We speculate that this is true because the positive regulation of mitochondrial biogenesis might not be as important for Puf3p in fermentative conditions as that in respiratory conditions: First, based on gene deletion data, the mitochondrial genes with P3E are significantly more important (having more severe growth defects after gene deletion) than those genes without P3E (*P* = 4×10^−6^) under non-fermentative conditions, but these two gene groups do not show obvious difference in deletion phenotype under fermentative conditions (Figure 5B). Second, severe growth defect after PUF3 gene deletion was observed in YPE, but not in YPD (Figure 5C). Therefore in non-fermentative condition Puf3p regulates both mitochondrial biogenesis and mRNA degradation, but in fermentative condition, it might only regulate mRNA degradation, albeit more efficiently in this condition. The expression difference of PUF3 in two growth conditions can also be explained by the fact that mitochondrial biogenesis in non-fermentative conditions is extremely important for yeast because the organism relies on respiration, and therefore mitochondria, to generate cellular energy in these conditions. In contrast, the function of mRNA degradation might not be as essential to the organism under fermentative conditions.

Although loss of a functional PUF3 gene shows a negligible effect on organism growth in YPD (Figure 5C), our results in this study indicate that Puf3p regulation of mitochondrial gene degradation might be important for yeast fermentative growth during evolution. After whole genome duplication, the post-WGD species (including budding yeast) evolved efficient fermentative ability [47]. Mitochondrial function became relaxed in these species [27], [28]. Most post-WGD yeast species can live even without a functional mitochondrial genome [47]. A large number of mitochondrial genes are down-regulated in yeast fermentative growth [48]. Degradation of mRNA by Puf3p may accelerate this gene-expression regulation process during environmental switches. Interestingly, our results showed that mitochondrial genes having P3E had significantly relaxed codon usage bias in the post-WGD species, which is not true for other mitochondrial genes.

### Evolution of the Puf3p post-transcriptional regulon and reuse of extant modules for novel functional roles

Understanding the origin of genetic novelties is a challenging issue in evolutionary biology. One of the prominent models proposed by Francois Jacob for evolution of genetic novelties in gene regulatory network is tinkering evolution, in which evolution reorganizes pre-existing networks to produce novelties [6], [49]–[52]. Our results provide an interesting paradigm for the evolution of post-transcriptional regulons that is consistent with this model. The Puf3p post-transcriptional regulon might have changed significantly at least twice in fungal evolution. First, although it is conserved in all the studied fungal species, the dedicated function of the Puf3p post-transcriptional regulon in mitochondrial biogenesis independently evolved in the *Saccharomycotina* subdivision. Second, although the regulation of mitochondrial mRNAs by Puf3p is conserved across the *Saccharomycotina* subdivision, the Puf3p target genes evolved a reduced codon usage bias in the post-WGD species, which might be consistent with the functional relaxation of mitochondrial genes in the post-WGD species due to the emergence of their fermentative life-style during evolution.

It was shown that although some microRNAs are highly conserved, their target networks can change dramatically during evolution [13]. Our results, together with those from previous studies, indicate that post-transcriptional regulons by RBP and microRNAs might share similar evolutionary patterns: *i.e.*, the interaction mechanisms between the regulators and their target genes are conserved, whereas the target network is plastic during evolution. As post-transcriptional regulation plays an important role in regulating gene expression, this evolutionary scenario involving post-transcriptional regulons could lead to significant gene-expression divergence among species.

## Materials and Methods

### Sequence data

Sequences for the forty-two sequenced fungal species were downloaded from the Fungal Comparative Genomics database [53] and the National Center for Biotechnology Information (http://www.ncbi.nlm.nih.gov/).

### Orthologous gene definition and motif detection

Using the InParanoid software package [54], orthologs between budding yeast and other fungal species were identified. The PUF3 orthologs in other species of fungi were identified manually based on the best alignment. Eight repeated motifs of PUF protein were detected by the SMART sequence analysis (http://smart.embl-heidelberg.de/).

Because the Puf3p binding motif in its target genes (P3E) is conserved between yeast and humans, based on the profile of P3E in budding yeast, we used a Perl script to detect the target locus of Puf3p by fixing all seven invariable sites and allowing flexibility in the fifth site (Figure 1B). For each species, we scanned 1,000 bp of DNA sequence downstream of all annotated genes to discern the occurrence of the P3E motif. The percentage of motif occurrences in each 50-bp window among all genes in each species was calculated. Multiple occurrences of the motif in the same sliding window were regarded as independent events. To see whether the motif occurrences in a species are different from random expectation, we calculated GC content of the 1,000bp downstream sequences for all genes in each species. Based on the observed GC content, 10,000 random sequences of 1,000bp were generated by a perl script and the occurrences of PUF3 motif were calculated. The significance of PUF3 motif occurrences in each sliding window was calculated by comparing its motif frequency against the frequencies of P3E in these random sequences by Fisher's exact test (more focused tests were also conducted in Figure S1). The Bonferroni correction was used to correct for multiple comparisons. Genes with P3E in their 250bp 3′ downstream regions were defined as the target of Puf3p.

### Functional enrichment analysis

The sub-localization information for genes in budding yeast was downloaded from the Saccharomyces Genome Database (http://www.yeastgenome.org/) [39]. Based on the identified orthologous genes between each studied fungal species and budding yeast, genes in each species are classified into different localization categories based on the sub-cellular localization of their orthologs in the budding yeast. The hypergeometric test was used to test the enrichment of genes with P3E in each localization category. The Bonferroni correction was used to correct for multiple comparisons.

### Codon usage bias calculation

According to the InParanoid results, we identified all the orthologous clusters that contained the known cytoplasmic ribosomal protein genes in *S. cerevisiae*, regardless of gene copy number in each species. The ribosomal protein genes in each species were used as references to calculate the codon adaptation index (CAI) value for each individual gene in the same species by CodonW (http://codonw.sourceforge.net/) [55]. In order to compare codon usage bias between different species, CAI values in each species were standardized so that the mean and standard deviation were 0 and 1, respectively.

### Microarray data analysis

#### Gene expression in fermentative conditions

Microarray data were downloaded from (http://www-genome.stanford.edu/yeast_stress). The expression profiles of mitochondrial genes during cell growth in fermentative (YPD) media were used to do the analysis. We counted the number of the up-regulated and down-regulated genes (defined as their expression level was higher or lower by 1.5 folds than the reference pool, respectively). Fisher's exact test was used to assess whether the number of up or down-regulated genes were significantly different between mitochondrial genes with P3E and without P3E.

#### Gene co-regulation analysis

The collected microarray data under different experimental conditions were downloaded from (http://www.weizmann.ac.il/home/barkai/Rewiring/) [29]. The dataset compiles microarray gene expression under multiple conditions and contains 1,011 data points for each gene. Correlation coefficients of gene expression among the studied mitochondrial genes were calculated in R.

### Quantitative real-time PCR

Cells were grown in the YPD and YPE media until optical densities at 600 nm reaches 1. Total RNA was extracted using the Trizol protocol [56] and cDNA was synthesized using an Invitrogen kit (Cat. No.18080-051). Using the ACT1 gene as reference, the expressional levels of PUF3 in fermentative and non-fermentative conditions were measured by quantitative real-time PCR.

### Gene deletion analysis and growth measurement

The rate of growth for deletion mutants were downloaded from [57]. Two-tails student t test was used to compare the average fitness contribution of mitochondrial genes with and without P3E in fermentative and non-fermentative growth conditions.

The deletion of the *PUF3* gene was conducted in the BY4741 strain (*Mat* **a** *his3*Δ*1 leu2*Δ*0 met15*Δ*0 ura3*Δ*0*) by homologous recombination and *ura*- was used as the selection marker (the primer sequences for gene deletion are available upon request). The mutant and the wild type strains were grown overnight in YPD (2% glucose) and YPE (3% ethanol) media. Cells were then transferred into fresh media and grown until the optical density (600 nm) reached 0.2. Then 4ul of growth media were dotted onto the YPD and YPE plates with ten-fold dilutions. YPD and YPE plates were incubated at 30°C for 48 and 72h, respectively.

## Supporting Information

Figure S1 The occurrences of the P3E at the downstream of all annotated genes in each species. (A) The randomly generated sequences based on the GC contents of 250 bp regions after the stop codon were used as background to calculate motif enrichment; (B) the randomly generated sequences based on the GC contents in each sliding window were used as background to calculate motif enrichment. Red denotes significant enrichment of the observed P3E motif in the real sequences in comparison to the random sequences.
(0.21 MB TIF)

Figure S2 The occurrence of P3E for genes functioning in mitochondria in yeast species. The PUF3 motif profile from Gerber et al, (2004) [17], which has two extra nucleotides at the 5′ of the motif in the paper, was used. As shown in the figure, species in *Saccharomycotina* subdivision have significantly higher percentages of mitochondrial genes that have this motif than the other species. The hypergeometric test was used to test the enrichment of mitochondrial genes with P3E in each species. The *P* value is smaller than 1×10^−8^ for each species in the Saccharomycotina subdivision, but larger than 0.2 in each of the other species.
(0.74 MB TIF)

Table S1 The forty-two sequenced fungal species used in this study.
(0.09 MB DOC)
