# Supplementary figures and images for: Tinkering Evolution of Post-Transcriptional RNA Regulons: Puf3p in Fungi as an Example
Source: PLoS Genet. 2010 Jul 22;6(7):e1001030. doi: 10.1371/journal.pgen.1001030 (PMC2908677; doi:10.1371/journal.pgen.1001030)

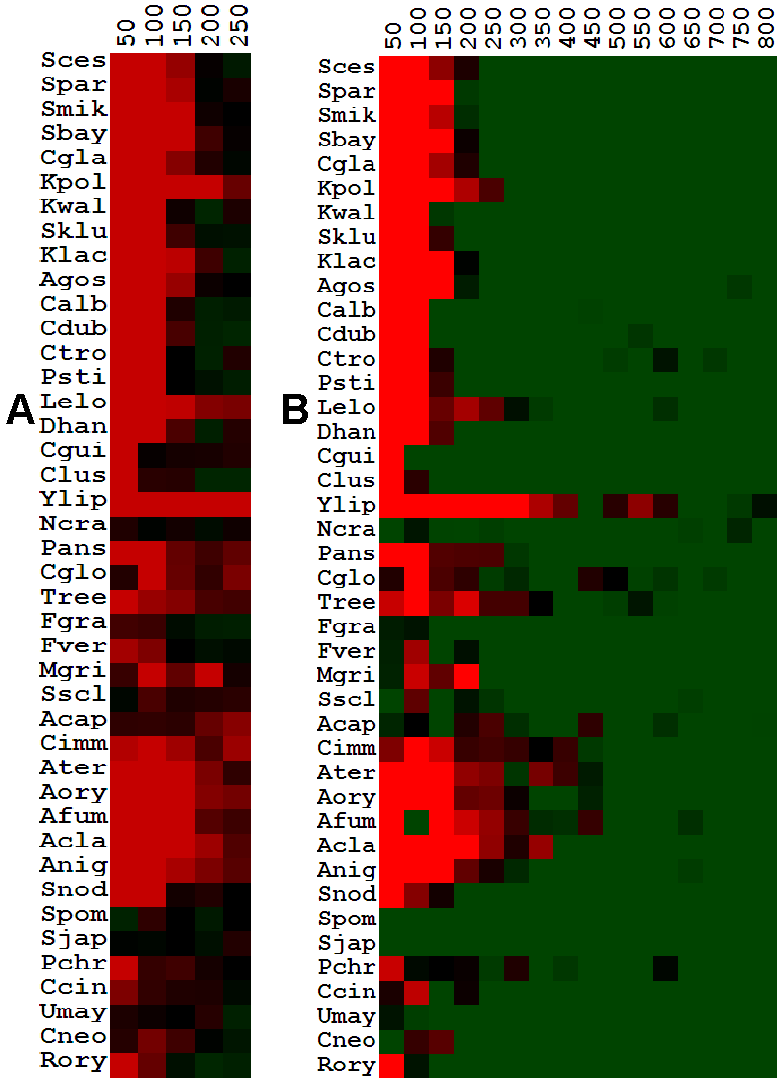

Supplement: Figure S1 — The occurrences of the P3E at the downstream of all annotated genes in each species. (A) The randomly generated sequences based on the GC contents of 250 bp regions after the stop codon were used as background to calculate motif enrichment; (B) the randomly generated sequences based on the GC contents in each sliding window were used as background to calculate motif enrichment. Red denotes significant enrichment of the observed P3E motif in the real sequences in comparison to the random sequences. (0.21 MB TIF) [file pgen.1001030.s001.tif]

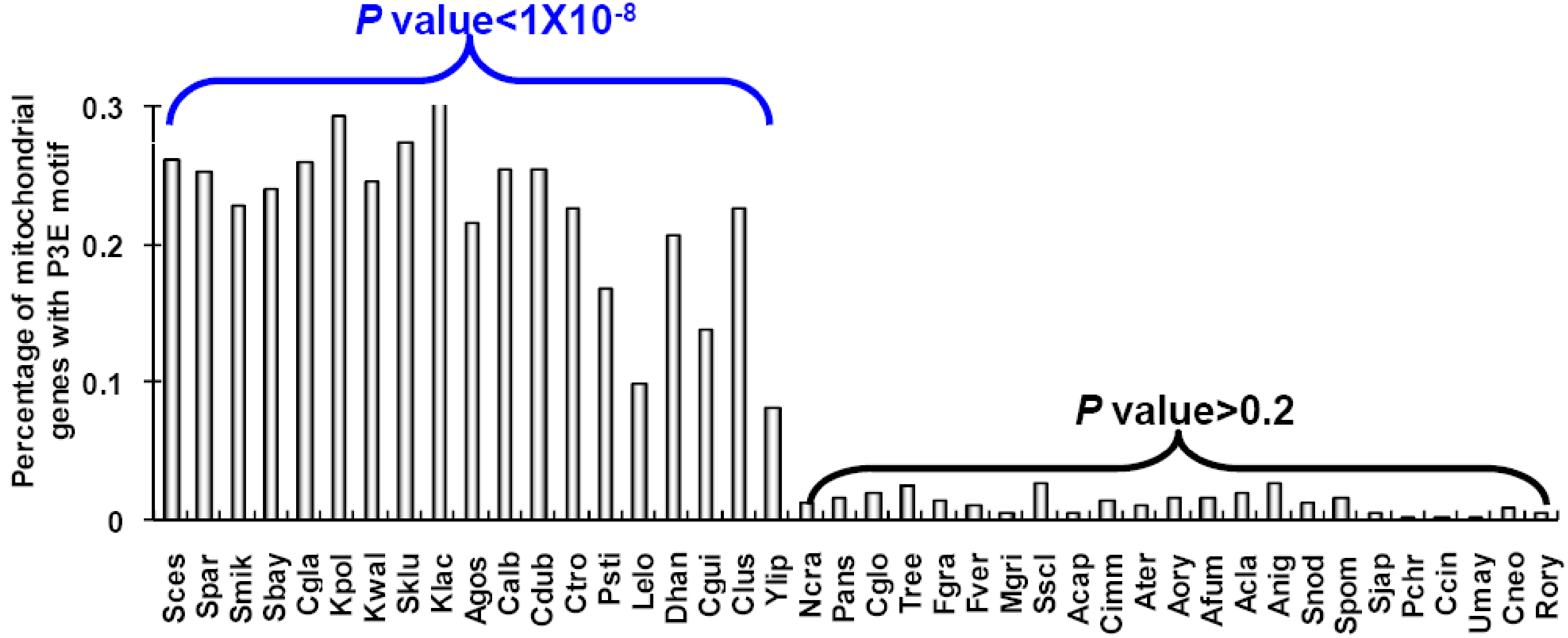

Supplement: Figure S2 — The occurrence of P3E for genes functioning in mitochondria in yeast species. The PUF3 motif profile from Gerber et al, (2004) [17], which has two extra nucleotides at the 5′ of the motif in the paper, was used. As shown in the figure, species in Saccharomycotina subdivision have significantly higher percentages of mitochondrial genes that have this motif than the other species. The hypergeometric test was used to test the enrichment of mitochondrial genes with P3E in each species. The P value is smaller than 1×10^−8^ for each species in the Saccharomycotina subdivision, but larger than 0.2 in each of the other species. (0.74 MB TIF) [file pgen.1001030.s002.tif]
