# Supplementary material for: Tinkering Evolution of Post-Transcriptional RNA Regulons: Puf3p in Fungi as an Example
Source: PLoS Genet. 2010 Jul 22;6(7):e1001030. doi: 10.1371/journal.pgen.1001030 (PMC2908677; doi:10.1371/journal.pgen.1001030)
Supplement: Table S1 — The forty-two sequenced fungal species used in this study. (0.09 MB DOC) [file pgen.1001030.s003.doc]

| **Orthologs of PUF3** | **Species** | **Short** |
| --- | --- | --- |
| anid_hcap-186R_2.75-g10.1 | *Ajellomyces capsulatus* | Acap |
| ACLA_096290 | *Aspergillus clavatus* | Acla |
| Afu6g04310 | *Aspergillus fumigatus* | Afum |
| AAL152W | *Ashbya gossypii* | Agos |
| est_fge1_pm_C_10386 | *Aspergillus niger* | Anig |
| AO090701000120 | *Aspergillus oryzae* | Aory |
| ater_1.12-g12.1 | *Aspergillus terreus* | Ater |
| CAL0000998 | *Candida albicans* | Calb |
| ccin_1.105-g46.1 | *Coprinopsis cinerea* | Ccin |
| cdub_11-g158.1 | *Candida dubliniensis* | Cdub |
| cgla_D-g221.1 | *Candida glabrata* | Cgla |
| cglo_1.2-g1082.1 | *Chaetomium globosum* | Cglo |
| cgui_1.7-g181.1 | *Candida guilliermondii* | Cgui |
| anid_cimm_1.183-g122.1 | *Coccidioides immitis* | Cimm |
| clus_1.5-g330.1 | *Clavispora lusitaniae* | Clus |
| CHROMOSOME2-g495.1 | *Cryptococcus neoformans* | Cneo |
| ctro_1.6-g54.1 | *Candida tropicalis* | Ctro |
| CAG86050.1 | *Debaryomyces hansenii* | Dhan |
| fgra_1.37-g26.1 | *Fusarium graminearum* | Fgra |
| fver_2.3-g260.1 | *Fusarium verticillioides* | Fver |
| CAG98483.1 | *Kluyveromyces lactis* | Klac |
| kpol04907 | *Kluyveromyces polyspora* | Kpol |
| kwal_211-g5.1 | *Kluyveromyces waltii* | Kwal |
| LELG_04613 | *Lodderomyces elongisporus* | Lelo |
| MG04985.4 | *Magnaporthe grisea* | Mgri |
| gz.6351 | *Neurospora crassa* | Ncra |
| pans_25-g6.1 | *Podospora anserina* | Pans |
| pchr_41-g45.1 | *Phanerochaete chrysosporium* | Pchr |
| Picst3-86424 | *Pichia stipitis* | Psti |
| rory_1.50-g2.1 | *Rhizopus oryzae* | Rory |
| sbay_c566-g3.1 | *Saccharomyces bayanus* | Sbay |
| YLL013C | *Saccharomyces cerevisiae* | Sces |
| SJAG_00686 | *Schizosaccharomyces japonicus* | Sjap |
| sklu_c2097-g2.1 | *Saccharomyces kluyveri* | Sklu |
| smik_1210-g1.1 | *Saccharomyces mikatae* | Smik |
| SNU01515.1 | *Stagonospora nodorum* | Snod |
| spar_52-g2.1 | *Saccharomyces paradoxus* | Spar |
| SPAC1687.22c | *Schizosaccharomyces pombe* | Spom |
| anid-sscl_1.1-g42.1 | *Sclerotinia sclerotiorum* | Sscl |
| tree_10-snap.179 | *Trichoderma reesei* | Tree |
| UM03431.1 | *Ustilago maydis* | Umay |
| CAG79436.1 | *Yarrowia lipolytica* | Ylip |

Note: protein sequences of PUF3 orthologs in each fungal species

>Clus_clus_1.5-g330.1

MPSETIWSNLSDPASPRVTSKFRSVFDQVDSNTGLMESRRFSFQETSELESNLFDFKNSLSQVPYAASTGGPHAPVTFATSKNAPIQLGTASISGGIANIHPPKDSFLQKFSSVADATRDIELSNSIATLSLDGGARRTSFNYENNVPLLNVSPHGSMNENLNIASSRGSRHQSISEKIDNFNNNSPIQTPAVLSSDLNNPGNNASGADASKAASKHPQSFWNPATATSFTPAATFNYFLENSPFPHVGAPIPPNPYPKSGPMMPPIHTPPFMMGNFMEGGVSNMMSGTEGTPESDSTSVSGVSETKKPTEPVQEGAPVTGSKLPTRNAGIPGIGLMSRQPHPSAAGYVFHPFSPYSMYTPTPQGVSPPPPPIEVRADGGSSAQGFVPPLSGQPHHGKRGKTGRGGHNGGKSSHHIYRSPLLEEVRSNPKSKEFYLKDIHGHVVEFTKDQHGSRFIQQKLPSATAEEKEMVFSEIQDISYDLMTDVFGNYVIQKFFEFGSDSQRQILLGYMKGNIHELSLQMYGCRVVQRALEAIPLEDQIEIVEELKDHVLSCAKDQNGNHVIQKSIEKIPFENVRFILDSLDSHIYHLSTHPYGCRVIQRLLEYSDIEDQQHILAELNRFLFYLIQDQYGNYVIQHILERGTPSEKEEIFEVAFSSIVNFSKHKFASNVIEKCIKHGTLEQRKRIWREVMLGNEDLEKETVADDSPLALMMKDQYANYVIQKLVECFHAKSKEKKDLVVKLRQYLKQLSMKNSYGKHLASVEKMIAVAETALVEADRS

>Cdub_cdub_11-g158.1

MQSETIWSSGHNSPIQNNPLLSSTTNNASQYKSVIDQVDPEVIRLFANSNTFDPLSSTTTTSGPGSGSGTGTVKVADRRLSFNDGSDIIESDFFGFKRGALSAITAPVVGNGNNGSHNHHNHNQVINKNFGTASISGGIANRHPPQDSFLQKFSSVADATREIELGRLSLDDKTNTNNNNIGVNEIKMVSTSAFTSPHGSLNENLNMPVPTRGGSRHQSISEKIDNYNNNSPIQTAAALSINSDLNSDGGNTNNNESYLNSTSVNANATKNQGNYSHNFWNPAAATSFTPVGPGVTIPTPNYFIDANGLPMPSMPMPPQGFYPPRGGDASNSGGANSISPPPPFMIPSPPPPFLDPSVYSMMYGGNFPQPPPPPPPSQQQQQQPQSQTNPGQENKGNETDSKSQDNKHSDTESHEEENDNTKDYQQGSYQHGPGPGPIGVGLMNRQFSPASFMFHPFNPYSMYQLSPPLVPPEAALGGMTSPPPQQLQQQPLPPPSSQGKSTSGVSGTSSSSTTTSHHQHSKYPHPHHNNNVHPHTNVNNTGVGSKRKGNFKGKNSSNNNNNNNGSGGNHIYRSPLLEEVRSNPKPYQLKDIHGHAIEFTKDQHGSRFIQQKLPEATEEEKETIFNEIWEISYELMTDVFGNYVIQKYFEYGTTTQTQVLLESMIGHIHELSLQMYGCRVVQRALEAIDNEGQLRIIEELKNHILICCKDQNGNHVIQKSIEKIKPFSQIRYILTSLDNQIYHLSTHPYGCRVIQRLLEYSDINDQKFILSQLNNFLYYLILDQYGNYVIQHILENGTSEEKEPILEIVLGSVVQFSKHKFASNVIEKCIKFGDTNQRQRILHEVMIGNEKMLSNNDDDNSDNDEPVKEDSPLALMVKDQFGNYVIQKLVEAFDGEERKLLIIKIKKCLSLSSNNLASIRNIRNIIDNVSESNQNLKVKS

>Cgla_cgla_D-g221.1

MMPNDGVWGSPADGAHADLLHSLSKTNSRTSDDHNMSTNSYLSLSSMAYGNNANQGHLVDASIDAELASIVSSLSALSHTNTNTMPQQQQQQASLLSSPNLQKIGTATSSNIGGFRRSSFTSDHNYGPEHIVSETPTASLSVGTAPPYYYSYSNQNNGGNTAGSAGNMQGLTTSLSSQRLNYLNSNQYSASIAGPILFHSNNNGSVSGQNNNQQMGANSGFFEKFGKTIVEGTKELESANYANDTDTIHSSKSYTSLHNNENTPVVEPIPNHQQAAYLSTSAIDDTASSSSSNSQLTLESPDYVNSNMNMRKGPGGVWKNFTDSQSFKPNHFGPLPYPNFQSPHPNFPMFNPFLPMGIQNHPPGLNNNNNVNINPANTNPGNDTVNRDPNLANTETQQEQINAQNLNENGDHNLNNSNASPYYYYPINIPNEAQQPINSNASTAPKKKRGGDKKALHKQQQGNMNPYLDSHHQLQRPFAQKKGQTPTSPPAARPMSPLSNQPINFYKKQQQPPSGQSAGTQRTTSSASNSSSQNSKNTANNNNNFHRSALLEELRANPTNTDLTLKSIYGHALEFCKDQHGSRFIQKELATAPPPERELVFNEIRDHALSLANDVFGNYVIQKFFEYGSKTQKDILVEQFRGKMEELSLQMYACRVIQRALEFIDAQQRIDLVRELSHCVLQMIKDQNGNHVIQKAIECIPIDLLPFILNSLEGHIYHLSTHSYGCRVVQRLLEFGTLEDQKRILEELKDFIPYLIQDQYGNYVIQHILQHGSDVNLASEHMRVIKQEIINNVADNIVEFSKHKFASNVVEKAIIYGTDDQKIQLMKMILPRDKEHAANLEEDSPLILMMRDQFANYVVQKLVVVSQGDDKKLIVVAIRAYLDKLNKSASSGNRHLASVEKLASLVESVEI

>Agos_AAL152W

METAAQAERIRGQMSTTEHTTDPWAGSVDYGSASRRNSNMDPELASIVSSLSALSTGPGAGAPQQLGGFRRASMNSNSSDVESELLFAGMSPTVRRTTLSVGGGAGGGGRLAMLGHYPASIAGTLPAGGQGGTFFERFGRTLAEATREVETGLGTYSVRQALRESAASSAQDLLARPGVAAATSDENALRKMSVSSETVESVSESMSMHNESNPSHNIWNVANAPVFRPNHGDSGGDPYSSVYGGFPYGMFGQYPSGTGLGGGGAFAAGGTVAKGEEASREEPQGDAATATNGQFAFPAGAPFLYFPVGSNGGSGAAIPAVLPPQPSRHSPSESQKGKSKGNPYLQQHPRGYKAPGMPLQNSNSKAAPQTSISGKGHHGHHYSSSKQSSTQNQHQANQQQSQYQHQSSSGATKRNGKNQQPIMRSPLLEEFRTNPTNKTYKLHEIYGSALEFCKDQHGSRFIQQELATASNIEKEVIFNEIRDHAIQLSHDVFGNYVIQKFFEFGTKTQKDILVEQFRGKLEALSLEMYACRVIQRAFEFIDEDQKIDLVMELSSSVLTMIKDQNGNHVIQKTIECIPMSKLPFILESLRGQIYHLSTHFYGCRVVQRLLEYGSKADQEEILNELDQFIPYLVQDQYGNYVIQHILQHGGDNPAENHIDKSKQDIVDTISKTVVEFSKHKFASNVVEKTILYGSASQKRQVLDKILPKDEEHAATLEDTSPLILMMRDQYANYVVQKLVGVGTGNDKKLIVIAIRSYLERLNKNNTLGNRHLASVEKLAALVEKV

KL*

>Sscl_anid-sscl_1.1-g42.1

MSSAAANRSAGTMNAQRSTRFGDFPTAPGRHNSDDRTSQNSTLGQTFGNGGTWQPSTGIWGSNTIGSGFANTKRDASRSRAADNDGFPDAPSGSGALAASSEADPWVARANGPWNPPDTTSPTLQSSHSGSTSPSHLRNSIPSQTPQSLLELQTYQQSRPAIGQGTSFSRQQPKSSLDPSSGSFKFSRKPSFGFNDDKENSSQFSSNQESAYDIDVSSRAFRMDQLGSQNPSYLGIGSVSRDGSMPPSRASESGLNNLTFGNGNPTHGSIGGHTPNNSIHSHRPSFSAMSGSFAQTNSSRYADLNTQTEAELREKFAGFGFTNDMDQTGASQIGNALSSSYSPTNQNFNQQAFQVNGGLAMWNDGSSGPKVNNYDHFSNQAPFTDQAYFTKGPRFDRGSVSPASSDYRRSLNSPKYFSGTPPTGPEQIYRPSSRAGPRIPQGPSELDRRLQHVQYSQHQAYLYNSGHFQGQYSPHLYDYSPQTFRQTNVPPYGYPIPLPPYPAQSIPTRPAKDQDVGLGVRSVLLEEFRSNAKSNKRYELKDIYQHVVEFSGDQHGSRFIQQKLETANSDEKEQLFREIQPNALQLMTDVFGNYVIQKLFEHGNQVQKRVLAEQMKNHVMELSMQMYGCRVVQKALEHVLADQQAELVKELEADVLKCVKDQNGNHVVQKAIERVPTEHIQFIIEAFRGQVHILATHPYGCRVIQRILEYCQPRDQERVLEELHQCASNLITDQYGNYVTQHVIQHGKPEDRAKIIKIVTAQLLTLSKHKFASNVVEKSIQFGTDEQRHTIVSLLTALHSDGTSPLQLMMKDQYGNYVIQKLLGQLKGAERAAFVEDLKPQLLALKKYNYGKQIAAIEKLIYGQDDSQQSYAASPTGTASGVHTQSIEMNSSAPTPLLTNGQNSPQSSSLPSANVSTIEDLTDSASSDKTAVAANEKSCPEV

VISGV

>Rory_rory_1.50-g2.1

MSPGGQKSDLHQPKAENQPQGQIAALLHGKLDYEDLFAGNSSSLFPDDHRSNSAPPTQQLMNPRRGVPDGFVDSTHRDSQDGRQPNAQRQLWQREEELGVQDPRAFNNDFPRTPSPLFALQQQARQRHTAVTASNSTNSGFSFDDPALVGDLDAIQKQYRDQLTALEYDDMMYRNRQSSSSSLASAHQDSSNNLPSMLHMTMDDNPISPPVRNKFAFPGTPPARPNSTPPGRNVYDDMDRQTEMLMNQFGGFGLNDEEEYNATTPNAQYMQQVQQASRFQSLSAVNSPYFGLGASPHLYNQQALYNTLPEDEGALGLDLSMYGRWSQGDTAYRRQQQQQAAVSAAANLSHLNGPLTAPAGIDPAGFPDNPLPNNATDKKLRALQQQHLLIQQQQQQILAARQQLLLQQQMQLNNSNVLKARSDKHHHNQHQQVQSSQDIAMSIRSPLLEEFRNSKNKKYELKDIDGHIVEFSGDQHGSRFIQQKLETANSDEKQMVFEEVLPNALQLMTDVFGNYVLQKFFEHGNQMQKTILAKQMEGHVLSLSLQMYGCRVVQKALEHVLTEQQAKLVKELDGCVLKCIKDQNGNHVIQKAIERVPAQHIQFIIDAFHGQVYNLATHPYGCRVIQRMFEHCTEDQTGPLLDELHRCTSQLVQDQYGNYVIQHILERGRPADKSLVIEKIRGHVLQLSKHKFASNVVEKCVDFGSKRDRQLLIEEVLQPRPDG

>Cglo_cglo_1.2-g1082.1

MATNSRRSRLSDYGSAPTDSHTFQPGLGNSIFNSPASGPWNNNTVGPFANTGRVVAPKDAKEFFASAGLGIPPALETAVGHEAWRTSYWVIADSKQARTLPDTASPRGRSDASLHDPNQRQKFFHSPPAMTQRGPIGSKPTTAAAVDGSKGAFKYPLKFSSYADEGEDGQSSNQSFEHRFAALPASLNAVGSGPSRKSISGLAEPDLPTQADSFNDFAFGIPTLPSMHSQRPSIDESSLPTHPGMGSFDNAAQNFQFNPVSQPWDNGNGYGNGFVKDGYPNGTSLEKRGSIAGRNSPAGSTYRNGGSLNSPRSFTGATQPNPDGWSRPTSRDPGLAAELARRGLTDQFAQHPASGFFPNTIFPQNYQQYPPPYAAYLEPAHNAHLAGYPVPTPYSFAPTGVPTRPARDQDPGKSLRSVLLHEFKHSPKSKKWELKDIWSHVVEFSGDQQASRFIQQKLETANSDERDQVFAEIEPNAVQLMKDVFGNYVMQKLFEYGDQVQKKVLANAMKGKVVDLSMQPYACRVVQKAFEHILVDQQTELVKELESEVIKVAKDQHGNHVIQQAIVLVPREHIDCMMAGLNGHIYELAAHQYGCRVVQRVLERGTETDKAAVMSELHDSAELLITDMYGNYVIQHVLEKGRPEDRGRMISVITPQLLTLSRHKNASNVVEKCILLGTPEEQRSIRDQLMGDDANSPLFQLMKDQFGNYVIREDRAVLVNKLASHLQSLRQSGATNKQIEAMDRLVADSQAPASVATSHSHNFTPASTTPTSPGLHVDVSSVAPTPNLTMDPSSPLSTPSSGPPYLNGDTNESMSGQPAGKETTHSQ

>Sklu_sklu_c2097-g2.1

MDSELASIVSSLSALSNPSAQHGQPHQLSQAQGHQGQLGGFRRPSINSNNGSDVESEILFQNQTSPVLRRTTLSVGGASVGAPNGVNNRLNLLGHYSASITGGHPVTNQQQLQQGGFFEIFGRTLVEGTREVELNLGTTSGRASRRESVNAMDSLARATTNATIQGSEVSVPRERRVSISSDALESVSENLNMQDNKNAARRNIWNVANAPVFKPQGADEEQPQNPEIFNSMYTFPYGPFQQFGGPLFAPMMSPPVPSPHPTIHTPAVQDGGELDDTDDIGKNDAQRQHQQQQQQMPPQFVFPGNPYVFFPSGATPPPHPVPAPPQSASPVSTIESQQNTSNNSNGSHANVRTHGNPYLHHPRGSRLNNTGPPQFKPSTPQQQAAPSQQNNKPKGKNNVPRSALLEEFRNNPTNKTYRLSDIYGSALEFCKDQHGSRFIQQELVNASDAEKEVIFNEIRDEAIALADDVFGNYVIQKFFEHGTKTQKDVLVEQFTGKMEQLSLEMYACRVIQRAFECIEEQQKVALVQELSHCVLHMIKDQNGNHVIQKAIERIPIDKLPFILGSLNEQIYHLSTHSYGCRVIQRLLEYGSLKDQDQILDELDQFIPYLIQDQYGNYVIQHILQHGGEHTNIHIGSTKQNIVDIVSKSVVEYSKHKFASNVVEKTMLFGSDSQKRQIMDRILPKSLDHAAHLEDNAPLILMMRDQYANYVVQKLVGVAQGEDKKLIVVAIRSYLDRLNKVNALGNRHLASVEKLAALVEKVEI

>Sces_YLL013C

MEMNMDMDMDMELASIVSSLSALSHSNNNGGQAAAAGIVNGGAAGSQQIGGFRRSSFTTANEVDSEILLLHGSSESSPIFKKTALSVGTAPPFSTNSKKFFGNGGNYYQYRSTDTASLSSASYNNYHTHHTAANLGKNNKVNHLLGQYSASIAGPVYYNGNDNNNSGGEGFFEKFGKSLIDGTRELESQDRPDAVNTQSQFISKSVSNASLDTQNTFEQNVESDKNFNKLNRNTTNSGSLYHSSSNSGSSASLESENAHYPKRNIWNVANTPVFRPSNNPAAVGATNVALPNQQDGPANNNFPPYMNGFPPNQFHQGPHYQNFPNYLIGSPSNFISQMISVQIPANEDTEDSNGKKKKKANRPSSVSSPSSPPNNSPFPFAYPNPMMFMPPPPLSAPQQQQQQQQQQQQEDQQQQQQQENPYIYYPTPNPIPVKMPKDEKTFKKRNNKNHPANNSNNANKQANPYLENSIPTKNTSKKNASSKSNESTANNHKSHSHSHPHSQSLQQQQQTYHRSPLLEQLRNSSSDKNSNSNMSLKDIFGHSLEFCKDQHGSRFIQRELATSPASEKEVIFNEIRDDAIELSNDVFGNYVIQKFFEFGSKIQKNTLVDQFKGNMKQLSLQMYACRVIQKALEYIDSNQRIELVLELSDSVLQMIKDQNGNHVIQKAIETIPIEKLPFILSSLTGHIYHLSTHSYGCRVIQRLLEFGSSEDQESILNELKDFIPYLIQDQYGNYVIQYVLQQDQFTNKEMVDIKQEIIETVANNVVEYSKHKFASNVVEKSILYGSKNQKDLIISKILPRDKNHALNLEDDSPMILMIKDQFANYVIQKLVNVSEGEGKKLIVIAIRAYLDKLNKSNSLGNRHLASVEKLAALVENAEV

>Sbay_sbay_c566-g3.1

MEMDMDMDMELASIVSSLSALSNGNNNGGQAAAVVGGAAAGSQQIGGFRRSSFTTSNDIDSDILLLHGSSESSPIFKKTALSVGTAPPFSTNSKKFFGNNSNYYQYRSNDTASLSSASYNNYQTNNAAGNLGKNNKVNHLLGQYSASIAGPVYYNGNGHDSSNVGGGGFFEKFGKSLIDGTRELESQDRPEHAANQPQFITKTVSNASLDTQTTFEQSVRSESNTNTNKMNTNTTNSGSFYHSSSNSDSSASLESDQNAHYPQRNIWNVANTPVFRPSNNPAVPGAPNMVAPNQQDGLANNNFPSYFPPNQFHQGPHYQNFPNYLIGSPSNFISQMISVQVPANEDTGDFDGKKKKKSNRPSSSSTPSSPPNTTPFPYAYPNQMMFMPPPPPPSAPQQQQQQQDQQEHQENPYIYYPSPAPKQQEPSDKFRQQQRQHEQTSKPLSRNFATAKNSSKKNSSFKSNEPVANNHKSQSHSHSQTPPQQPQQPTYHRSPLLEQLRNSSSDKNSNSNLSLKDIFGHSLEFCKDQHGSRFIQRELATSPASEKEVIFNDIRGDAIELSNDVFGNYVIQKFFEFGTKIQKDALVEQFKGHMKQLSLQMYACRVIQKALEFIDSQQRIQLVVELSDSVLQMIKDQNGNHVIQKAIETIPLKKLPFVLNSLTGHIYHLSTHSYGCRVIQRLLEFGSSEDQCNILNELKDFIPYLIQDQYGNYVIQYILQQDQFTNKEMVDVKQEIVETVADNVVEYSKHKFASNVVEKSILYGSKDQKKLIMSKILPRDKNHALNLEDDSPMILMIKDQFANYVIQKLVNVSEGEGKKLIVVAIRAYLDKLNKSNSLGNRHLASVEKLAALVENAEV

>Pchr_pchr_41-g45.1

MRSPQSASFYDYGASGRVPSQYYYPPQPMMYHAPPPTATPAGNGHHKRRSMQAPYQTSPLMYSSPRSGTTARPQSYHAGTTPMTLHGHSMYPGMQMPSPHLGRGRRHEDLNAGFRSPLLEEFRNAKDRKWALKDIYGHVVEFSTDQHGSRFIQQKIETADEEEKQIIFDEIMPQNALKLIQDVFGNYVIQKFFEHGNELQKNLLAKAMEGHVLPLSLQMYGCRVAIEHVSPEQQSVFVAELADNVLRCVKDANGNHVIQRLIESVPPERLTFVTSFQGYVCDLATHPYGCRVLQRCFENLPDHQTRALLMELQEHALQLMQDQFGNYVIQFVLEHGQPQDRAIIVCKLQGQMLHMSRHKFASNVVEKALVTAESSSRRALIDEIMALRPDGSSPVVSMMKDQFANYVLQRALTVADQDQKEALVDLVKPQLQNMRKFSHHGRHLVAIERLIQKCTPTATRERTPAPAETTGAPAATDSVITPSSPN

>Pans_pans_25-g6.1

MAGTTRPSRFPNLAPSSTGNGANGIEKSSQPITNTFPAPNTWGSGTSIWSNTNTIGNSFVKSREAVGARDSNDGFVTPSGSGALASTSEIDPWGRPGGPWNSNDNSQNRNVSGQTSPNRTRSEVQLHDMSGSMPYYSGSQTMTQRALGGSKDAKNGFPKYTSQYADFTDDKDNSFSNLNGEPEQTVGRYPASRSMQDQSFLGNGHSRDHLSTSGQSDNDLHGQGAPYADYPFGTPHTSIHSQRPSLNGPTGSFHGQNPRSYDHNNMGQQIPDDDLPERLGRMAIGSGLNGGSNALGNLQSFGNGSQDFQLNPGSQPWDHGQGYQAGHSRDSYSNSISLDRRGSGVDRSSPAGSTYRAGATGVGLNSPRSFTPAIDSWSRPVSRDLRSGPEADRRSLSHFPPQSPYYQNGYYNFPQYAPGPYDAVYGRQPMQFPGYPLPQYPFAHNGVPPVRPSSDRDPARGVRSLKLEEFKTGNKSSKRFELKDIYGFIVEFSGDQHGSRFIQNKLETANSDDKNQVFHEIEPNAIVLMKDLFGNYVIQKFFEHGNQAQKQVLAAAMKGKVVELSMQMYACRVVQKALSHVLVEQQAELVKELEPEILTIVKDQNGNHVVQKIIQTVPRQHIGFIFDCFRGRVSELSSHAYGCRVIQRALEHGNEADKQSIMKELHSCAQMLIMDQYGNYVTQHVITDGSPDDRSKMVALVMSQLPIFSKHKFASNVVEKCIKHGTADQQRDIRDRFMSRGDDGNSFLVSLTKDQFGNYVLQTLLSELQGQDRDVLVNEVRPLLASIKKMCTGKQIAGVDRLHNAITSYTPSSTAPTSPGLHVDVSSAVPTPNLTMGPNSPSSSPPSTSESAVEETIGQTDAKPATTDATVNIQDQADEV

>Kwal_kwal_211-g5.1

MSTTDEQRDPWAAHDVEFGAGSVHGANMDSELASIVSSLSALSGVSANQQHGQIGSFRRGSFHSNQGSDVESEILFQSQTSPLLKRTTLSVGGVSLGAPSGVNNRLSKLKNYSGSLSGGSVLQQQQAGHGGFFERFGQSLAEGTREVELNLGRSSGRASRRESMNAMESLSRVTTNSTFHGVTEGRRPSASSDALESLSENLNMQPEDSSKLNHRNIWKVAEAPVFRPQAAESSGGEPVFQQPPIEMFNQPYGFPYPNYWKMGAPIIPPAMPAPEGDKELPDAAAARPQEPLNAPVESGTQQPFPPFIYGGNPYMFFQPGAVPPQPPIFPPPANGHKETPKGSHSKSHGNPYLYNNRNYKPPRGSPGTSSISPQPHSSAPPSNGPPRGKGKNSVPRSPLLEEFRNNTNGKNYKLPDIYGSALEFCKDQHGSRFIQQELAKASDAEKEVIFNEIKEEIITLADDVFGNYVIQKYFEYGLKTHRDILFEHFKGKMEKLSLQMYACRVIQRALECIDEEQKVILVEELSGCVLQMIKDQNGNHVIQKAIERIPIARLPFILKSLEGQTYHLSTHSYGCRVVQRLLEYGSTDDQNIILNDLDQFIPFLIQDQYGNYVIQHILKQGPDTSDSHMGRTKQAIVDAVCKSIVEYSKHKFASNVVEKTMVFGSPAQKRQFMEKVLPRDVNHAVNLEDNAPLILMMRDQYANYVVQKLVGVAEGSDKKVTVVAIRSYLEKLNKINALGNRHLASVEKLAAIVENVEV

>Anig_est_fge1_pm_C_10386

MAFKNGLSERLDELRFPSPRSPPSESPFPGYNSLSPGHSNFVSAFSRPSGDVRGNLQRRFTTDSSKLASWSHLNHLGSSSQLPAPDHLDLLSSFEKKRQHIEYMREQRRRFEEDMKLLDMQHEKEKQDLERIAENLAKVGMSGPVSEPTTPPEYRENSYPAFTRPTRFSTSSVTSSPGFFNVFAPAQVTTPPSQVNHNSAQTPTNRFSVHSVPGSRRNSEKEDFTQEPTSPFRPGPSIHRYSMPSTGIGSQIRPTLSGFNSSSGLESFNASKYLFHNEDDRATLKDEDRIPTPDFKSILKLTDTDDKFPTLSRRDDSGLLSANSDALDLANSRTPNPETWNTHSRHRTTHQSMPQNALNMFRQLGNQTDENHAHSSNSARHAARHSLEANLLYSAEEHSENLTATASSRPASLQSSYSTNDLPTVKGEPFNPAVTPPKTHAEQIQHNANLGRIPAVNPVNSRQQRDSPERDEAKLQGTRAQQTTLQASATPFGPPITTGPANSNSTVAPTTLTPFQAPFYGYGIQAYMGAPVQVSGQLQNYNAAAPFAGYAPYGTYRMPEGSAKSVTSRRSGDNDSAQLSRFTNFPLEHYKGELYGLCKDQHGCRYLQRKLEERNPEHVQMIFEETRLHVVELMTDPFGNYLCQKLLEYSNDEQRTDLINNAAHQLVKIALNQHGTRALQKMIEFISTAEQTQTVIHSLEDHVVELVQDLNGNHVIQKCLNRLSAEDAQFIYDAVGANCVVVGTHRHGCCVLQRCIDHASGDQKARLIAQITANAFALVQDPFGNYVVQYILDLAEPHFTEPLCQNFRGNIPALSKQKFSSNVIEKCLRTADFQIRRQMIDEMLAGAELEKMLRDSFANYVVQTAMDFADAETRARIVDCIRPILPSIRQTPHGRRIAGKMMASEGSGRGSAATSGQVTPNEMNSAQLPGPLQGPQKSFMYQHSPFPVGSQFGNQNFVPTAGSATGSNAPSGGPSEAIFASAVPQANGNLGAQSQLYAPSTLVVHGISATCKTTIVRNVLAALEVPHAIVRSPECITGRHLLTKILWATLEALGKRDEWEKYGKGRCEHVSSLAVLLGECLASLSDGSSNDKGKFVLVLDGIDKQREAPHTLLSALARLGEVIPSLCVVLILSSSPRPLFLQAAGVPHISFPPYTRKEAVTIILNAGPPAVSGLDDETASRLYPHFVSAIYDSLVGPTASSIPTFRSICEKIWPQFVAPITNGDIPPGGSNEWDFSRLLVKNRALFRHQGEGALVHHIVTEESAPANGSLSKPSMSAVSAPSPLPSLPYFPTLILTSAYLASHTPQRLDTIFFSKFSSSSLSARNKRAHHRRRLKVLSRAQAEDSREASRGPSTPSKKGKREKTRITKSTLESAFATSSATTSAVGSNAGITGPSTILTARPFPLERLIAIYHAIDPNPPANPIRLAAVSDAIYAELATLRRLRLVVPAAGRESGGRMGLGSGGLSSGNTTSDAGEKWCVNVSGDWIGELAKTVGVEVGEWLAGGLD

>Afum_Afu6g04310

MSGSSMNSDRSRGPHAGMGKGFGAGKSSWNTNIWGDSNLKSGFDDQNLAETGFEGKSGSGSLLPSSESDGWSSRPNLPWNTVNTTSASLSRGQNNGMTASPISTRPNDRSAPALSDTVDSTSYFSIPRSSGIGSSSGGGNHKTYLNAGADGISPCGDGLSFGNFGGLRSGDGRRHPNSSAFGGSPVGTGFPMKQGFTTLDTTRPDEITGSLTMSSLPQALPDTMSQPLARNGFAHASHNSASITSQRPTHASHPSFHSESQGFEGRFGGSSMDLSAEINTLQLNEGGFSGHPVSRPPYLSHSSYDGSLQRFKYQSAADESSYEAVGGYAGDGLSELPLGHHAGRSRLGEGSLSPTDLARMESTFYSALEAGAVPGSHYRNGSATRLSENQALALERKLRAMQHDQDLAHGAANSLQRIPYNTPAYDLAGYQAARLNALSGFYPVAQLGGLGSAGIIPRGHRDQDPAQVVRSPLLEEFRANSKGNKRYELKDIYNHIVEFSGDQHGSRFIQQKLETANSDEKEQVFREIQPNCLQLMTDVFGNYVVQKLFEHGNQSQKKILANQMKGHVLALSTQMYGCRVVQKALEHILTDQQASMVKELENHVLKCVRDQNGNHVIQKAIERVPSQYVQFIINAFKGQVSRLAAHPYGCRVIQRMLEHCEEVDRESILAELHACTAHLIPDQFGNYVIQHVIENGEEKDRSRMINVVLSQLLMYSKHKFASNVVEKSIEFGEESQRQQIISTLTSANERGESPLLGLMRDQYGNYVIQKVLGQLKGEEREGLIDQIRPLLSQLKKFSYGKQIVAIEKLIFDSTTLGANSLPPASSTTPPHSHKSSPQPSKRLVNDVENSRAPVVGAAPPTPPPTDNQGHTHGPAESKNLAKSTVTSLSVSESASADSNGSVPVSAST

>Ccin_ccin_1.105-g46.1

MAGQGLPFLPASNALSPVIQPPIQVDGSPSILPEMPTLLRQAPMLPPPPKELEAQPSELLQPGCSKLLLEFKTNLKKRWELKDVRGHIAEFSRDQRASRFIQQVIEDADTDALDLIWSEVASDDLLTISFNACGNYVVQKLLDRGSEAQRVKLATALQGHVVQVSQDAYGCWVIQKVLDVVPNHVRGQIVLEAEPHILTLVKDPNGNHVVQKILQVVPARYLTFVDAFHGRAVEIARDNYGCRVLQRCLQHLPFEAVQPLLQELKPFILEMICDQFGNYVIQHILQDGKTSEKEEIFHQIRGRVLRLARHKYASNVLEKALTHAPPLIRHAIIEEMLTTVKGFPKGVWQLMNDQYGNYVLQKALTLAEEPQRTVLSMATKCQLSNVRAAGKARAKQMASIQQILQGLEELRC

>Spom_SPAC1687.22c

MFTAVNSNPNASESISGNSAFNFPSAPVSSLDTNNYGQRRPSLLSGTSPTSSFFNSSMISSNYTFPHGSNKQASLESPVSYSNPIPSLTWLSLDGDSPDSLVSTPTAPSANHHGNPFPNGKQSIKAMPSLVNLQEDSVISKFPNSLEVPFRKRSESTSSSLSGLHSDLRPLKTELYGQLNSECGARFPQTLKSPLTPIGGDSARTVSASTARTSDKFFPRHTRAHSDFWIPATSKPSRHASHSSIGDLTTITQSSISSGSGSFKPSWDGSFDSSLMAHQSYGTSPAFANGNSPTLKNDSSFFGSASVRPTVSPIGTSFRQSLPDISAFGIPKTETNPSEVVAPGTIPISVLPTSNFSAATPANPSLINQNGQEFLQQSRVLYLFHANKQRHFELSDILGNVVLFSTDQHGSRFIQQKLATATEEEREAVFQEIASTSCLQLMMDIFGNYVVQKYFEFGNEKQKQILLSQIKGHVFSLSLQMYGCRVVQKAIEYISPEHQVQLIQELDGHVLDCVCDQNGNHVIQKAIECIDTGHLQFILRALRPQIHVLSAHPYGCRVIQRAIEHCHSERKLIIEELLPHILKLTQDQYGNYVVQHILRTGSESDKKYIFDLMIDHLLFLSCHKFASNVVERCISYISDVDRRRILNKIISEKAENCSILMLMMKDKYANYVIQKLLDASPEEERDLLISYIYPHISVLKKFTYGKHLIMSVERFRQKSISAVPKLASKECK

>Cneo_CHROMOSOME2-g495.1

MPHQETFLQQNERKKDYHLRSTQGPMSRISQNSGSNFRQDFNPSFQGNFYGQGNLSFHAAHTGPYGLHPAPHFVALTGFPPQFSSQYPGRPASTRKFDDPGVVRSALLEDFRLNKMKKWELNDIFGHIVEFAGDQHGSRFIQQKLEIATPEDRQKLFDEIYPNAYQLMTDVFGNYVTQKMFEHGDQLQKAALAKKMDGRVLQLSMQMYGCRVVQKALDHLLNEQRAKIVAELEPHILECVKSSNANHVVQRMINIGPPQSIPDSFIGHVEELAKHPYGCRVLQKTFENLDDKMKRSLLDEMHKCVISLTEDQFGNYVIQSVITVGKSEDRNKVVDQLKGRITTFARHKFASNVVEKALIHADPADRRALIDELIGMQPDGTNQVGMLLRDAYANFPLQTGMFAAEPAQREVLLEIVLPLLPPLRHTPVGKRIEGRLAQMENEYNISSVMMRKTLSSSTATTDTNAALCMSRTASGSTVPTSPELSTIHIPIIKSPKKDDIEGW

>Sjap_SJAG_00686

MGQLSEFRASDPNAYSQKLGFKTESEALHAWKLERNGMGVSNEWHFPSISQMGSHSTTVSPSAATVTNAFFPSPPPSALPQDYHDVSGMFGNGPRQSLTEPSSIDSGFLRSSQSSSSLSGFTPTHTTVLHPFSSVDLNGKGSVGSQDGKMNSFSRLSVDENVFRKSSSSSLIGQGPASSIGSLPSAKLSSSYKGNLNNFLGYRIPNSAPILFDHGIHSQSVSLPPTQNDFPSPLGEPATNSHKMFPLEQQQHSSRSEDLLYSPSNTAAAFNSSLLSRHNSLRRRSSLIAGSSLSQSNKQVHSPLEYWPPSSQGMSQGIPAQSRSLSYNFSPEHQAFMTSPTEQPTPSDIVATSPYHSSLNSPFASANTFQSKLPDSSGLSNIGSAVAFGPNGTSHHSKFRPHVAEQHQPNFHFGNPTDEIFELESKSASAPIRAHSPTSAGLSNAVGHLVLSGNSSSFMSSKQVSYSSEEKQSDSATDYTTRNKILHDIRNNKHKKLEIKQLVGHLAAFSTDQHGSRFLQQKIETCSDEDRALLFHDIVNGNCLQLMMDVFGNYVVQKLLEFGTDEQREVFTEKMKGHVLTLSLQMYGCRVAQKALEHIPLNRQVELIQELDGDVLKCVKDQNGNHVIQKAIECIPYGHLQFVVDAVMPNVYNLSSHPYGCRVIQRIIEHFADARSSVYLQLHTQILHLAQDQYGNYVIQHLMKKGSPSEQREIVEVVLGNVLHLSRHKFASNVVERCISYCSDTDRERFFNSLLGENEDGDTYLLNLIKDKYANYVIQKLIDVSKPELRDRIITVLNPHLNVLKNYTYGKHLYLVVEKFQRGNAEEPSENSKTVMTNMP

>Tree_tree_10-snap.179

MPSGSASNAQQPVGSSSFTGSNLYPNPSIWSPLAQSNTGGRSNGIGAYLDSALPGRAPSRGRIAAWRAPCILQCLKDANDLTSNGTESEENPTGSSALNANSEAELWAASSSRPWNTENPARSVNNSPPRARDAGIPTTSGLFSNSHPAAVGMRSSSNLVDESSQYSFAAAQRRAPQDGAYNDAMAAFAQSRENSLPPSRQSQGSPGYADMMFQLGQYQQGIGHDNTLAFQTKQLPHNPLSLPQAANQRAYLSNRQVEDDLNLRMNRQLSMNDGSTGVQAFNSNYMPWGGESGVSRLGSGFGTNADSLQLSTIRRPSLDRVSPAPGGSAYRMESGSGSRGLAPAAEPWNNGSRPSSRGQRAAEHERRASGQHMSVTSNFPAQYYPSPYGFAQLPAGYTSYVETYTQSFRPMLPNYPVQPVHPGYGINSMSPARSSRDQDPNRGMRSALLDEFRINGNKSNKRYELRDIYNHAVEFSGDQHGSRFIQQKLETANSDEKEQIFREIEPNAVQLMKDVFGNYVIQKFFEYGSQLQKKILAEKMKGKVVDLSVQVYACRVVQKALEHILVEQQAALTRELEPEILRVIKDQNGNHVVQKIIELVPRQHIDFIMKAVRGQVTPLASHAYGCRVIQRLLEHGTEADKAEIMGELHASAQLLITDQYGNYVAQHVIQNGEPEDRERIIRLVMGQLLTLSKHKFASNVVEKCIEYGTPAQRTTIREQLTTAGPDGNNPLQQMMRDQFGNYVIQKLLGQLQGDEREALVEEIKPQFYTLKKSVASRQVQALEKLLGLNGAKSENSAQSDTNSLAEVPSLTHETNSPQTNSPPSIHASAVGVPSIGATGNKASGDPVDGMPVRVEDADS

>Fver_fver_2.3-g260.1

MATGAFDQSQRQPMHAQTNSSTNATPWDTLHTRNLFANENGERRGSNAFNVPTSAAFWTTSLATRSPSTSPSRMAQEHIPNSDLGNDQNLAGHFSQMGISKERSTAIYGNAFRYDSETFNVNGFGRTQDHRSNSSRQSQSSPSYRATHTASKSTQSQQQFPNHMSQGSNHQALNQKAFNHNNSQSLDATSLNFLGNVPGAFILDPNCQPWSNDASGQRVNGNSLDLSSEMMNLAIFSEHNASYAIGLDQYPQRYHHLGHNNSGYGAPYPYHNQNNGPGPKIYKNQWVKEFCGRLTKRSGPQPELCEAYGHMVDASGEQESSRWLQTKLANATNEEKTRILLEIADDARTVMICSFGNYVMQNLIEYTGQGEKFHILQQMKGHVNDLARNKHGCRVVQKAIEHFLVDQNLELVQEIQPHLLDLMKHETGNHVIQKFVQELPLAHLTSFVRVAEEHALELSQDSHGCRVIQRLLEVCQEDDIRKVLDPLYPSMEMLATNQFGNYVVQAIIEHRPGSDRDRIVEMVVNKLLYFSKNKISSNVVEKCIAFGSDEQRTQIREQLCTVSANGKDTLFELINDQFANYVIKSLVHNTKGPGQQQLAQKIHTHLETMKKSAPLPKPSHSLSQVVEKILVVDNMSISLKIEVDSAEPTPMLTNETNSPQSDGLPSANGSTIGVPSAGIRKPEMAVRVHSDEA

>Psti_Picst3-86424

MPSETIWSSGTGSPIQASSSQSQYRSVLDLVDPEVAKLLSSSNERRFSFNDGSDIDSGIFGLKRGSFSAITAPVGGVSGYHNGANSVGGGASGQRINFGTASISGGIASRHPPQDSFLQKFSSVADATREIELSNGLSKISLDSHGHSGNSRRTSFNNQAGSQNEPIKQLSEIGVLSSPHGSLNENLNMPAPRSSRHQSISEKIDQYNNTSPIQASAALLINSDLNLNQDHQSVNQQQPSNVPHNFWNPATATSFTPDSSYNYFMDGSSLPGGVLVPPPGPPQNFNPYGGRSGNIPPISPPPFMIPLPPPQFMDPGLYNMMNFPPANNVSNAESELAKEITAASQSSSSSAPNAASDSEEKLEEHKESGSGRNVHTPGIGILGRQQIPLAQFMFHGFNPYMYQQSPPPMGITPLSPNQHPSESSMFVEGIPPPPAPPAAAKDSAPTPPASSGKRGRNGAKNSNAGKGGNHI

YRSPLLEEVRSNAKGKEYYLKDIYGHAVEFTKDQHGSRFIQQKLPDASDEEKEVIFNEIRDISYDLMTDVFGNYVIQKYFEHGSTTQKKVLLDYMIGHIYELSLQMYGCRVVQRALEAIDLDGQIKIIEELRDYILICAKDQNGNHVIQKSIERIPFDRIRFILDSLDNQIYHLSTHPYGCRVIQRLLEYSNVEDQQVILQELNRFIFYLIQDQYGNYVMQHILERGEPDDREAILKVVLGSVVNFSKHKFASNVIEKCIKYGTLSQRKRILREVMLGNEDFNVELVSDESPLALMMKDQYANYVIQKLVEGFDANSEEKRILVVKLRQYLKQISSKNTYGKHLASVEKMIIVAETALIEAENN

>Klac_CAG98483.1

MSLSEQLNGWNTAVSDDKDNNTQIDSELASIVSSLSALSNPNVQQQAQQQVQQQQVQAQQQQQIGGFRRASFNSNTGSDVDSEIFFNTQNSPMLRRTTLSVGGMSLPENSSSHAQNRLNAMNHYSASIAGGLTAQFQNNNNPNLNSYNGSVTNSNSIGVNNSSVTSGFFERFGRALAEGTREVELNVGVGSVNVGGPPSSSNHAGTGSRRASATTGLEHLSRVGSNTTLHGARRMSDTSEVLDSVSESLSMQNNEPNSTTIWNVAAAPVFRPQNAEHMQQQYHYQQQQQQQQSPLHQRQPQFQPQFSYRRSKDGKTAPFPSDQDIDPNDPASFANQQAAPFNYGYPGFNQFGMPMFLPPVLSPPPQPFPGMNGSNDNSQDVETTDGVTKEASASRKNQHESELSETSGETGNHPTMMPHPMAPYPFPSPYPFMYAPIKEDDTENNKDSETVKPPLSPTFLPPNPYMFMPPSMQPRPGNLQDAPSSPPSADNGSGSNKRNNDSKYSGKNKSNPYLHAGKAQYSQFSPPPSRAGMAVPPNTLQSSSGSGANKQGRQGKNARQNNKQPIVRSPLLEEFRNNSSNKVYKLSDIYGSALEFCKDQHGSRFIQQELATASDADKEVIFNEIRDQCIPLSHDVFGNYVIQKFFEHGTKTQREVLVDQFRGKMENLSLEMYACRVIQKAFEFLNEDQKVDLVSELSHCVLAMIKDQNGNHVIQKAIECIPIEKLPFILQSLRGQIYHLSTHSYGCRVVQRLLEFGTLKDQDDILNDLDEFIPFLIQDQYGNYVIQHILQHGTEDTSSHIGMSKQNIIDIIRKNVVEYSKHKFASNVVEKSVVYGSKNQIRQILDQILPRDEEHAADLEDNAPLILMMRDQYANYVVQKLVGVATGEDERLIVISIRSYLDKSNKNNTLGNRHLASVEKLATLVEKIQI

>Ctro_ctro_1.6-g54.1

MYQQSPPLMAHEAALRGMTPPPGMGMPPPPSQMDNSPTYNNTGASQGQQQQQQQQQPQSQQQPQSQQQPPQQPSTLKGDNAKHEGAGSKKKSNHKGKNGNGGNHIYRSPLLEDVRANPKAFSLKDIYGHAVEFTKDQHGSRFIQQKLPEASDEEKQTIFNEIWEISYDLMTDVFGNYVIQKYFEHGNSTQKQVLLESMIGHIHELSLQMYGCRVVQRALEAIETEGQLRIIEELKDHILVCCKDQNGNHVIQKSIEKIKPFSKIRFILTSLDTQIYHLSTHPYGCRVIQRLLEFSDEDDQKMILTQLNNFLYYLILDQYGNYVIQHILENGTPEEKEPILEIVLGSVVQFSKHKFASNVIEKCIKFGDLNQRKRILHEVMLGNEDINDDSEIGDDSPLALMVKDQFGNYVIQKLVEAFDGEERRLLIVKIKKCLSLSGNNLASIRNIRNIIDHVSENEFK

>Ncra_gz.6351

MSPNTSPNMGRGRAPRGFPPYRPENNLHNQPHRHSFDVDFKIWSMPNASAEVGDRPPSTSSGSNDWSYSYSNQTAHTRNGTNGSSPRTSVPPGLAYRLDGISSSMRRPSHDNGFGAMEHASERLSVGGFNVASSGPSEPAERQGLGLTPYAAQHSHRSSVVADSSTLSVNWQNNRASVGDLDAAQTNVPWLARKPAEVTDTDRPMFSSNLWSNSSHTVAASARPFTPASPTAWENGSGNGLQTSNGVQTGNGFQSGNGFQAGNGFQTSNGFQTGYNANGFGNYTGAQQSRSNFSQAPSRREFSSPTNIDRRASTQSFIQQPQPTTNLPFDTVTLQQQLQMYNMAHNLGMAGAYSNPSLPTTGLAMGAMSSGSMQSGKLREYLNTRNAPQKWDLKQIYGSIADFAADRAGSRFIQDKLQSASSEEKAEVWRELMEELMPLMTDVYGNYVVQKFFEHGTQEQKTSMAGIIKKNMLRLSENKYGCRVVQKALDNIFRRYQVELVNELKDHVDKLNKSQEGNHVIQMIIKLLPRDEIGFIYDSFRGPGKVMELALNQYACRVIQRALEHGNEEDRLYLVSELHKGAHTLITDAYGNYVAQHIIEAGKPEDRARMIAAVMSQTITLSTHKHASNVVEKCINYGTPEDVRRIRDMFFSPQDGMGGNEEQQFFIDTLEPKINELLKNHKGLDERQRNALKRFQGIINELRKDIDKKEELAKNGSSAPPSLDPASPSLHISSALPTPDGGSEPNSPLDLGMTSSANTTSPIGSADGNGSNGKQSLLVVNINGGLDATDAFAQLHIH

>Fgra_fgra_1.37-g26.1

MSSDDSINRYSMPVTRSRTGLYDVGLDQTNTTRFLFGDEDSNALGHSVPDENFPTLVRRDDQMRPQQNLSSINGTSNIVSDLVGLASRPASLRHSIDLKYISENAIETGSLMSPPGNGNMATPPKLQGSFSSNDVPTVKSPGGSSSKANTHAQQHFHNHNASLGRIPAGAIHRGHSREISSDNTAVSREQSGYPSIQSALQASAAPFGPSTTAPAPASMGNTPTGAPSMNGNFNNNSGYYPVSGYGVPQGAPQGSVPQGAPQGATHQPSAYNANMLASSMQQMSMNGANGGSMYQPQNYNGYNAGHYNQSNQPRDSQARVMQHRRQLDNEAMSRFQNMPLESFVGTIYELCKDQHGCRYLQKKLEERNPDQVHMIWNETNKHVIELMTDPFGNYLCQKLLEFCNDDERTTLIQNASQDMVRIALNQHGTRALQKMIEYVSTPQQVHIIIEALRFRVVELIQDLNGNHVIQKCLNRLPPQDAQFIFDAVGNNCVEVGTHRHGCCVLQRCIDHASGDQKLWLIQRITEHARVLVQDPFGNYVVQYIIDLNEPIFTEPIVQTFKDCISQLSRHKFSSNVIEKCLRCSQPPSRDLIVDELLRNQDMERLLRDSFANYVIQTALEYATPHYKYRLVEAIRPILPQIRTTPYGRRIQAKISAFDNRGSAASSGQVTPADNTQGQIPLRAAHSRGLSGNVPMLQGNGIPPSGPMPTMRQNMAVYSPTPAMNGQTPPAGAPVQQPQYGQAPGDFTPNSASNGAAKGSAGPTTPANGSTGNTGEVQWV

>Umay_UM03431.1

MPRKEQATSVLDASLTTAVESNPDSISLSESTPSILPASGPETAATSSSQSPVHYPADADEAKLDADHASEADIQARIEKKRLEHEKQRAMHQKAFEQQMALLEKKQREEEQNLLSQRSSSNAGAAASAPTTPPNEALTPDPKSAVAPNLNNAKPLSHLYQSTPSSRRPSADAQCSPDQLASAVGAMSITAKPAVDAAAKDKGENFTPVFSEQFLFDDELDNEDSAFVKKYNLKANDDQFPILVQRNAEARNASRGADWPSFGGSNANTSGAADASADATGDTTSSSLRGSIKSKSPPPAPATPSMAASFGTSAGRQPSAFPLPSPSPHLSQTGRSSPAINATTFAGVASRQASGPGSPSPGLSGLGGDMARSGGGSRFVNTAVTIPSAAANLKNQTMAAAAAAGAGASAGAGAGAGAAGSAAFPGFNLGAFSGASTGSESPNRFGTYSPNSFDAAAVADARRASRPSSGYYDAFNPTAGANAPGSLFPPDKYSMGMVGADEHAKLGGLGPHGSGGGPTAALVKHARGKGELDATTQLDDLQGDIFALCKDQHGCRFLQKKLEEGNPAHRDMIFSETFTHFAELMTDPFGNYLCQKMLEYCTDEQRNLIVELVAPELVTISLNMHGTRAVQKMIDFLSTPRQIHSIIVALSMNVVTLIKDLNGNHVVQKCLNRLGAEDNQFIYNAVAAHCVEVATHRHGCCVLQRCIDHASEAQRVQLVAEITYNALTLVQDPFGNYVVQYVLDLSIPRFTDAVVRQFVGNVCLLSVQKFSSNVIEKCIRVSEPGVRKQLIEELLNRTRLEKLLRDSFANYVVQTSLDYADPVQRMRLVECIRPILPVIRNTPYGKRIQSKLQRDNLDLGPPNGVPYSVLHAAHQQQLHAMAVMAGGGGARGNAGYMGMNAYGPPPPGGPMTGPPPAHHAMHQAIHHLGAHQPQPHAMYGGAGARHAAPAPNLPPPNFGQAGGYAGGFTGSLAPPMGMQQGGGGGGRGGGGGGGDGGGGGGYANKAGVAGAGAGAGANGGFQDTDWRCSCSLQVCFGRTSGHDDLSLAFPFSFTYCSG

>Cimm_anid_cimm_1.183-g122.1

MDCGQVGGQSRAFGTAKASWKNDVWGNGHLTDAFSDGTRENGHFRASKSMSDEMMEGKSGSSCLLATSESDGWDGRANMPWNVSPSHGHSLTPPGIDAQSRDSSPPYFSASRPAAIGTAVKSSAQRAFFTQSDPQSVAVSSVGTPSGSTGYMDHNTPPRNINPAAFKNSNLGARFGSGATLPLTNSDMGSSDNLNGSLGFRPFQAANVGLAGKSTGPYTHLSRNSMSSFSQRPAHSSHSSFHSDSDGNDIREPRARYDIAHEFSKLGLEGNSYVLHSHSNSHRSAYMGPSFDGSVPHFKSPFGDEVSGSVLRAYSPEPFSDMSAYQSIPRSRHGERGMGSPSLNDYVRNTNKGFYSANGTPPAAPRLVISPGNRLAGHLADEQTELLDRKLRSLQHEQQEYLQSASPISSRRALQQAQGYGLSSYHAAQINQVGNPYAMATFSGLPTVVARNQYREQDASQSLRSPLLEEFRANNKGNKRYELKDIYNHIVEFSGDQHGSRFIQQKLETANSDEKERVFQEIKPNAIQLMMDVFGNYVIQKLFEHGNQAQKKALAQQMMGHILNLSTQMYGCRVVQKALEHVLLDQQAAMVKELENQVIKCVKDQNGNHVIQKAIERVPQAHIQFIINDFSGQIQRWAVHSYGCRVIQRMLEHCNEADRDAILGELHLCSASLIPDQFGNYVIQHVIENGRERDRSQMIAVVISQLVLFSKHKFASNVVEKTLEFGRPNDRSEILRIFTTPNERGESPLEGLMKDQFGNYVIQKVLQVLKGDEYQTLVDKIVPLLSHLKKHSHGKQIAAIEKHLAKPTPPTSSAASEGNHLCRGNEVDSSYEDNGLSTGGTPSSRGSTASSTNTSIDPADIAESKSAPAPSTHSSRSA

>Aory_AO090701000120

MGGERNRGSHGGMGQPFGGGKASWKNTIWGDNLDQHAGDKAFEGKAGSSSLLSSSESDGWNGRPNMPWSTVNTSSNVLSRATNNSMTTSPIQTRAGDRGTGSLAEAGDSSYLTLPRSAAIGGTAGSTNHKAYLNTGSEESRRHANSSAFGGSPVGSGFPMKPGFSTPLESTRPDEMPSMSALPQGLPETVAPTLGRNSYTHASHNSASFAPQRPVHSSYPSFHSESQGFEGRYAGGPADINSGLSKLHFNEGSYAAHPSSTRPGYLSHPSFDGSFQRLKYQGDEPAYQGASYASEGASDVQLGYQAPRNRVGDNPISPTEYARVDSPLYAALDSGSVHVPHYNSASARLSDAQAAALERRLREQELAQQAINPLQRLPFTPYDLARYQGSGMNALSGFYPVAQLGAAALASRGHRDHDPSQTVRSPVLEEFRANSKGNKRYELKDIYNHVVEFSGDQHGSRFIQQKLETANSDEKEQVFREIQGDSLQLMTDVFGNYVVQKLFEHGNQTQKKILANQMKGHILALSTQMYGCRVVQKALEHILTDQQASMVKELEHHVLRCVRDQNGNHVIQKAIERVPSEHVQFVINAFIGQVEKLATHPYGCRVIQRMLEHCKEEDREAILAELHVCTAKLIPDQFGNYVIQHVIENGEDKDRTRMVTIVMSNLLTYSKHKFASNVVEKSIEFGQESQRHQIISMLTSTDDNGENPLLGLIRDQFGNYVIQKVLCQLKGAERDALVEEIKPLLSQLKKYSYGKQIAAIEKLVADSNSPTNGTLPHTTSTTPPNSHKSSPQPSKRAVNGLDGCRAPVVGAAPPTPPPIDPQSNGDGSSDTKTVTKSTPLTAAESAGTTPTTSVEINGAN

>Mgri_MG04985.4

MSNAGDKAGQTLGGGISYQGPDWKRQNIWSSSLGSFPKSQPPTSRGNDENGQAGAGALAAKSDVDSWSRRGPWETADNSQRKTSGATSPTRTRDSFNDMHASNTFTTSRPPINQGPGFSLNRQRQSAFDTTSNAFRYTSMTSDETESPSGYPISGGFGGDSKPLNMRADSRNGYSGGQAGTADSLQGYENAYAGHVSGMAHAGRPQPHSSSSFPAQSGPTRAYSTTQLDQQDLHESFKRSVTINDASDTGTSNFQAARSFQFNPGSQPWDSNGVATFKPGHNGYSIPEAHQEGIISDYSNGKRGSIPDRNSPGLNMRTNLASPRHMSEASIIPNGWGSRPASRDPRNGLENERRGSTNSFMSSTHSHAIHLPGQQQPSFYPPHPYYAQTIPSAYPTQLYDQYGNGFRTPLPFPQFGLPYSPYGHTGLPMAPSHGIKSQDPISGGRSRLLDEFRATSKSAKKYELKDIYNYVVEFSGDQHGSRFIQSKLETANSDEKDQIFKELEPNAVQLMKDVFGNYVIQKFFEHGNQVQKKALASQMKGKMALEHVLVEQQAELVKELEVEIVRIIKDANGNHVVQKIIELVPRQYISFVMDSIRGQVIQLSQHNYGCRVIQRMMEHGSDADKATIMHELHQHAPMLTTDPYGNYVIQHIITHGKPEDRQKVISIVLGQIVLLSKHKLASNVVERCIVSGTAEDRTAIRKIITTPGIDGTSPLQLMMKDQYANYVVQKLLEKLNGAERQAFVEEMKPQFNSLKKVSNGRQIAAIDRLMSAVGTGSGAAAGLQVDVNSAAPTPNLTMEPNTPQSTSPPSTDSSTHDDVTSDESNGGAEKTQSPTAGACPQVRIDEA

>Ater_ater_1.12-g12.1

MDKLLAKLSPKPQALGEPAIENEEIPRKISEQQAVVDQKKYALIGGMEADSSSVIPRPSTPQEARSSTDEAGSKQGPFPTDTPEVIRLKRELLAANSKIALQEQELAQTRVIKHTLDQALGPPSEADFGGREITEQTISHLQSAFNASNPSFNQLPDGWSGQDDSQSDISDALSAGAYNRTRGFWVPPTQQVFGMGLNAPPVDKPYGDPFPLPSNATLQESNRFWNTRTPNPTVSGQGFQSARVLSGPSLGACSIDAQLPGDQPRTLEGPPPLQRRLTTQVNRTGDWFSTQSSPWSAFTPIPQTRNPPRSPVNRQDSTYQQVGLYPIPPYHQQPVGTPLSPTANEFTSTHLNMVPWGTASTSGSHTQTYVSPLEPINYRRLLDKNVSCDWRYIVDKIVCNNDQQASIFLQQKLKVGTTEQKFEIIEAIVHQAYPLMVNRFGNFLVQRCFEHGTPEQIVAIANAIKGNTLSLSMDPFGCHVVQKAFDCVPEEQKAVMVHELLRRIPETVIHRYACHVWQKLFELRWSGEPPQIMAKVNEALRGMWHEVALGETGSLVVQNIFENCVEDEKVNGPTSDHMLMAKPGACRPAIEEVLAKIDVLAHGQFGNWCIQHICEHGAPHDKSRAIKHILLWSVDYSMDQFASKIVEKCLKIGGSEFLDRYLARVCTGRSDRPRMPLIDIAGDQYGNYLIQWILMNAAPHQRELVASHIRLLALDQALEFRSAGLATSTTIGSQPKGSMADVIVVEANGVLAMHRSDEGDTATVDACLDTVETARCLKYRESVTLLYSSYVLKHDRDLRNW

>Dhan_CAG86050.1

MSSETIWSNNGTSPLQSSTGTNQYRSVLDQVDPEVAKMLANSNSNPMISGDNDGNSRRFSFNDGSDMDGNFFGFKKGSLSAITAPAGGYAGSNNGSNTGPQRINFGAASISGGIASRHPPQDSFLQKFSSVADATRDIELSNNLGKISLDAPSSRRTSFNNTDASNNMTPLGEVGGILGTPHGSLNENLNMPVPRTSRHQSISEKIDNYNNSSPIQAGAALSVNSDLNSNANDQTSAIRHPQAPHTFWNPATATSFTPNNSYGYFMENNGLQGAPVPPQNFNPYNRNGPSAPPISPPPFMIPSPPAQFMDPGLYNMMTYGINDDSPDGEHQEDKDNSDDAKNGDLNNRLETKQKDVSRKGHTPGIGLMNRQAGAGAGFMFQPFNPYSLYQQSSPPVGMSPVADPNSGNSLLDDGTSKNVPPTQLTRPSHAAPSNGNKRKGNPRNASSSGKGTNHIYRSPLLEEIRSNFKGKEYFLKDIYGHAVEFTKDQHGSRFIQQKLPESSDEEKEVIFNEIRDISYDLMTDVFGNYVIQKYFEHGSPIQKKILLDCMIGHIYELSLQTFGCRVVQRALEAIDLDGQIQIIEELKDYILVCAKDQNGNHVIQKSIETIPFDRIEFVLESLDNQIYHLSTHPYGCRVIQRLLEHSDAEDRKKILGELNRFIFYLIQDQYGNYVMQHTLERGNPEDREEILKIVLGSVVNFSKHKFASNVIEKCIKFGTLDQRRRILHEVMIGNEDYNVETVSDDSALALMMKDQYANYVIQKLVEGFDAKSDEKKILVVKLRQYLKQISSKNNYGKHLASVEKMIIVAETALVEAERST

>Acap_anid_hcap-186R_2.75-g10.1

MALALEDAQRSGVQPSTIGTSSTLKDTDPSKPSPASSIGKTFGTPKTNWNSNIWGNNSLGGGFGDPAIDKGQRRVDHKGRDPNPELPIEGKTGSGSLLSTSESDGWSGRNNSPWDLSNSTSQLRGPLANPPRQVLQNDHSTPMGISNTSGVTSASYFSVSQSSTIGHPSHTPNNMSFITHPDIFPSSPAQDNSAVMGITTYRSDEAGRRRMSCVPLKNNLGASFPHKQGLSLCDMDAPLSENAINSLNLQSFSPNGTDQAPGSKEHSPYGHFSHSSVSVVPQRPAHSAHASFHSDSHGAGPRYTGSQSDLISGVGKLQMQDEAYYNFNPNILAQRPSYHSSTSYDPSLNHFQSSNLNSNLSSHGAGVLAPDDTIDIPAKHSFNELRLTDRGPASPRVNDFARNVHNFYSAGGTPIPNGQFCTSSGSRLTGQLPDGQTELLERKLCGLQQEQQDYLKSATNPIPGRHPLSHGYRFSGSPGVSRVNALSNYYPVTSFGGLGSSSILPLNFHREQDPIQVVRSPLLEEFRTNSKNNKRYELKDIYNHIVEFSGDQHGSRFIQHMLESANSDEKDQVFREIQPNCLQLMTDVFGNYVVQKLFEHGNQSQKKILANQMKGHILALSTQMYGCRVVQKALEHILTDQQASMVKELENHVLKCVKDQNGNHVIQKAVERVPSVHIQFIINAFKGQVHRLAAHPYGCRVIQRMLEHCTEPDRRAILEELHACTSSLIPDQFGNYVIQHVIGNGEEHDKARIISIVISQLLVFSKHKFASNVVEKSIEFGADDQRAEILRQLTTPNDRGESPLLGLMRDQYGNYVIQKVLGQLNGLEREALVKKIEPQLTQLKKFSYGKQIAAIEKLIYDSHTTDGHSDSLSTHRKTSSRLSLSELNTSPSSTDDSGTPTSPSLGTQSSRSSSLPSTTTSDVEGSMDQRKQNIVFSAVTTPMSECNEGLKVPALENPDLKFAH

>Cgui_cgui_1.7-g181.1

MFSEDPFSGNSEAQGRRFSFNDGSDIDDSIFGLKRGSLSAVTAPAGNSRDVMPSGPRPPFGTASISGGIANRHPPQESFLQKFSSVADATRDIELSNGLGSLALGSESRRTSFNSAAEKPSGIGYLSTPQGSMNENLNSRNSRHQSISDKIDKYNSTSPIQGAASLSINSDLNGGSNSPKHQPNEQKQGHSFWDPAGATSFTPSNVGPFVPNNYSYFMADGVGMPLPPFMYTRAAMDGAPPAPYMVPSPPPFMDSQFYGMMRPPQVPVDNEEDDSSKEKSQETPSNAEDEPSIRGSHAPGIGLNRGIPTSFPYPFHPYPYQASPPPFTPSEVNQKVEERPASTPSQSSGKRKGGTRNFSSGKGGNHIYRSPLLEEVRSNSKGKEYHLKDIYGHAVEFTKDQHGSRFIQQKLPTASNEEKEVIFNEIRETSYELMTDVFGNYVIQKYFEYGNNTQKQVLLKFMIGHIYELSLQMYGCRVVQRALEAVDLKGQILIIDELRDHILVCAKDQNGNHVIQKSIEKIPFSEITFIMDSLEDQIYHLSTHPYGCRVIQRLLEYADPKRQQEMLDELNRFIFYLIQDQYGNYVMQHILERGSSKDREAILEVVLGSVVNFSKHKFASNVIEKCIKFGTVKQKRRILDEVMLGNEDPTVETVSDESPLALMMKDQYANYVIQKLVEGFDAKGEEKRMLVMKLRQYLKQISSKNTYGKHLASVEKMIVVAETALIDADNDP

>Calb_CAL0000998

MSSETIWSSGHNSPIQNNLSLASTTTNNTSQYKSVIDQVDPDVIRLFANSNSFDPLSSSTGPGTTGHTSGLTSGTDRRLSFNDGSDIIESDFFGFKRGALSAITAPVVGNGNNGRHHNQVTNKNFGTASISGGIANRHPPQDSFLQKFSSVADATREIELGRLSLDDRNNGNNQVSSVNEIKMVNTSAFTSPHGSLNENLNMPVPTRGGSRHQSISEKIDNYNNNSPIQTAAALSINSDLNSDTVNINSTTTNSNHDATKNQGNYSHNFWNPAAATSFTPVGAIPTPNYFIDANGLPIPPLPMPPQGFYPPRGGNASNSGGANSISPPPPFMIPSPPPPFLDPSVYSMMYGGNFPPPPPPPPSQQQQQQQPSQQQQPSHSNQDKKANETNSKSKETKYSDTESHEEDNEKQDNYHGPGPVPGPGPGPGPIGVGLMNRQFSPASFMFHPFNPYSMYQSSPPLVPPEAALGGMASPPPPPPLPQQQQPQQQSAPSSQGKSTSGVSGSSTSTSTSHHSQSKHHHHHNNNNNNNNNNNSSGSTPGVGSKRKGNFKGKNNTTNGGGNHIYRSPLLEEVRSNPKPYQLKDIYGHAIEFTKDQHGSRFIQQKLPEATEEEKETIFNEIWEISYELMTDVFGNYVIQKYFEYGTTTQKQVLLESMIGHIHELSLQMYGCRVVQRALEAIDNEGQLRIIEELKDHILICCKDQNGNHVIQKSIEKIKPFSQIRYILTSLDNQIYHLSTHPYGCRVIQRLLEYSDIDDQKLILSQLNNFLYYLILDQYGNYVIQHILENGTQEEKEPILEIVLGSVVQFSKHKFASNVIEKCIKFGDINQRKRILHEVMLGNETILGDDDIDGEPVKEDSPLALMVKDQFGNYVIQKLVEAFDGDERKLLIIKIKKCLSLSSNNLASIRNIRNIIDHVSESNSDLKVES

>Acla_ACLA_096290

MGTSSMNSERSRGAHAGMSKGFAGGKSSWNPNIWGDSNLGNGFDDQNLAEAAFEGKSGSSSLLSSSESDGWNTRPNLPWTTVNTTAAALSRAPNNGMATSPVATRANDRSAPALAETADSSSYFSLSRSAGIGSSSGAGSHKPYLNSGTEGISPSGDGLSFGNFGGLRSGDGRRHAGASAFAGSPVGTGFPMKPGFTSPLDTTRADEAAAMSGLPQTLPETMTQPLARNSYAHASHNSASFTSQRPGHSSHPSFHSESQGFQGRYGAASMDLSAGLNKLQLNEGGFSAHSAVGRPGYLSHSSYDASLQRLKYQNSGDESNYDAVTGYSGEGAPELPLAYQAGRSRLGDGSISPTEYARVEAPFYAALDAGAIPETHYRNGSGGRLTESQALALERKLRGMHQDQDLSQLPANPLQRIPLAPAYDLTGYQAARLNALSGFYPVAHLSGLGPAAIIPRNHREQDPAQVVRSPLLEEFRANSKGNKRYELKDIYNHIVEFSGDQHGSRFIQQKLETANSDEKEQVFREIQPNCLQLMTDVFGNYVVQKLFEHGNQSQKKILANQMKGHVLALSTQMYGCRVVQKALEHILTDQQASMVKELENHVLKCVRDQNGNHVIQKAIERVPSQYVQFIINAFKGQVNRLAAHPYGCRVIQRMLEHCEEEDRESILAELHACTTHLIPDQFGNYVIQHVIENGEEKDRSRMITIVLSQLLVYSKHKFASNVVEKSIEFGEESQRRQIISTLTSPNDRGESPLLGLMRDQYGNYVIQKVLGQLKGEEREGLIEQIRPLLSQLKKFSYGKQIVAIEKLIFDSPAAASASLSQVASSTTPPHSHKSSPQPSKRLVSDLEGGRVPAVGAAPPTPPPTDAQSQGDDGSSDSKTLAKSTLTPLSESESAGAVPNASIQVAGST

>Lelo_LELG_04613

MTDVFGNYVIQKYFEHDSKIQKLILLSHMVGHIYELSLQMYGCRVVQRALESLEDVDDQMKIIKELRDYILICSKDQNGNHVIQKSIEKIHPFDKIRFILTSLENQIYHLSTHSYGCRVVQRLLEYSNKEDQKMIMQELNKYIYYLIQDQYGNYVIQHILEQGTPAEKEEVLTIVLGNVVTFSKHKFASNVIEKCIKHGDVQQRKRILHEVMLGNEAEDDIKNSKDNGGENVEVSDDSPLALMMKDQYANYVIQKLVEVLDSNYPEKKQLVLKLRQYLKQLSDMNNFGGKHLASVEKMIMMAETAFDQN*

>Snod_SNU01515.1

MANRGGIALSNGDNERPSHSSHASSPWGNSIWNSTNTSSSLGFAFGNAKRDSSRPRENGNYDLTDGKTGSGSLVADSETEWRPSRPTWTDAGSSNNVHVRSSGVSPARKRSIAQTLPTQQYSDTSSTFYTGPRNSIVGTGPVSKPPKPLLDPTSTNFTSSRQVESLNASFSNFGFSQPDITQRPDTAVNSWPDSASVHSPNEDRRSVAPSEYFAPSSGAPSRNGSLPPSRHGAEPNQFNSMDAFSRLSQAAPRQASSFSYANGRSFQERSGSIQSDSFHTLNRMAMDQEQDARLAHRQSFSTNGQNMNYTHSGSEPHVARDSYPDVQLPTRTDDPRYRNGTYTPDSFANGHISDPTLQFQGFEFDNSRNAPNGSGVRQSPYHSQLHTPPVYDRLNPYSTGEQALSHPNNLALFQNKLAGYQIQQERRNYIPHSQFQQQQFQHILPATQLRHPYQYSYAVPNGVPMSAIPPHMAMATMQPMMPVQTPRGPREQQTTEGTTVQGAKLVEFRKESKTSKRWELPDIYDDVVEFAGDQHGSRFIQQKLETANSEVKERIFKELESNSLQLMQDVFGNYVIQKFFEHGDQTQKKILASKMKGHVSALANQMYACRVVQKALEHVLVDQQASMVKELEKDVLKTVKDQNGNHVIQKVIDRVPMEHIQVIVESFRGHIGVLAVNSYGCRVIQRLLEKVPEPQRRFIMTELHARGADLITDSYGNYVTQHVIEHGLPEDRAKIVSLITAQFLTFSKHKFASNVVERCLVCSDDEQRRELVNAFIAKNERGENNLLNLLKDGYGNYVIQKLLETLNRDDYNVFVAALKPELEKAKKLISGKQIVSVEKKMYRYDRVDSPTMPRDTNESNEAPPTPALSDSAQSPQTSSIPSTNTSTVDDPVHSATQSSQKETAIPISGVSIHETTS

>Spar_spar_52-g2.1

MEMDMDMDMELASIVSSLSALSNGNNNGGQAGAIVSGSAAGSQQIGGFRRSSFTTANEVDSEILLLHGSSESSPIFKKTALSVGTAPPFSTNSKKFFGNGGNYYQYRSNDTASLSSTSYSNYHTHHAAGNLGKNNKVNHLLGQYSASIAGPVYYNGNDNSNSGGEGFFEKFGKSLIDGTRELETQDRPEAAANQSQFIPKNVSNASLDTQSTFDQTAQSDTNFNKLNRNTTNSGSLYHSSSNSGSSASLESENAHYPKRNIWNVANTPVFRPGNNPALVGAPNVVLPNQQDGPANNNFPPYMNGFPPNQFHQGPHYQNFSNYLIGSPSNFISQMISVQVPANEDTEDSNGKKKKKANRPSSVSSPSSPPNNTPFPFAYPNPMMFIPPPPPSAPQQQQQQQQQQQQQQQQQQQQQQQQQQQQDQQQQQENPYIYYPSPNPVPVKVAKDEKNFKKRNNKNHSANNANNSNKQANPYLESFAASKNSSKKNTSSKSNEPGTNNHKSHSQSPPQQQQQQTYHRSPLLEQLRNSGSDKNSNSNMSLKDIFGHSLEFCKDQHGSRFIQRELATSPASEKEVIFNEIRNDAIELSNDVFGNYVIQKFFEFGSKIQKDTLVEQFKGNMKQLSLQMYACRVIQKALEFIDSNQRIELVLELSDSVLQMIKDQNGNHVIQKAIETIPIEKLPFILSSLTGHIYHLSTHSYGCRVIQRLLEFGSSEDQKNILNELKDFIPYLIQDQYGNYVIQYILQQDQFTNKEMVDIKQEIIETVANNVVEYSKHKFASNVVEKSILYGSKDQKNLIMSMILPRDKNHALNLEDGSPMILMIKDQFANYVIQKLVNVSEGEGKKLIVIAIRAYLDKLNKSNSLGNRHLASVEKLAALVENAEV

>Kpol_kpol04907

MSVTDPWAYSDFAPPSRGNSPILQNDNGVNNNKIDSELASIVSSLSALSNPGFGNTNNANNNNNNNNNNNSLGSSSHQIGSFRRSSVTTTTSGGSIANDDQNANPNMLDANDLSFLQKSLLNSANNRKSPMSVGTAPIMSGNNARMNILGHYSASINGPLLFSNSNNQQSAANEGFFEKFGKSLIEGTKELENSNLSASNSSTNIQDFANKNSMTLSVPTTTTAFRRASINSSVTDVTANTCSSSTSSNSNDRNTDILSEPNDEFNNRRNIWNLSNISNMPIFKPGFNNSNNSNNNNNNNQTHMFPYPQNNMNVGNPMMVPGQIPPPPNNPYYYMEGNYPPYMPNNIPMYFDPNMNTVNEMERHDSNKNKRNPYKKKNNGNSNNNNNNNHNVNNSSSNKGKHQINPYLDNKNSQSNLNVKSQKKQSLSPPTLPNSSQQSYVHQEKIGSVNSKNSNNNNSNNNNNSKLSNQQFHRSPLLEEFRNNSNNKKYTLKDIFGYVLEFCKDQHGSRFIQQELAVVTPSEREVIFNEIRDHILELSDDVFGNYVIQKFFEYGSETQKNILVDQFRNRMQKLSMQMYACRVIQRALEFIELQQRIDLVLELADCVLPMIKDQNGNHVIQKAIERIPIDKLPFILDSLKGQIYHLSTHAYGCRVIQRLLEFGSKDDQTRILEELHDFIPYLIQDQYGNYVIQHILQQKDEDLMKENMSPSIAKAKQEIVDIVSENVVEFSKHKFASNVVEKTILHGNEKQRNAVTSKIIPRDLEHAANLEDNAPMILMMRDQFANYVVQKLVSVTGGDEKKLIVVAIRAYLEKLNSSNSLGNRHLASVEKLANLVENVEV

>Ylip_CAG79436.1

MTGRAQSNNWDRSPPSRGIWGEYNKSHIVPYGPPTNTDTPTWSRAPSFQQERDLAPGGVGRKNSQGEEINPAYNPIRSGSFSGGISLIRSGNDIRRDSSMLPHPDHQFASKHFSFNDVEMQRPHLQAPEIFNPSQAHAPIPASGSSFLSRFSNIGDATRDAELNLGSRKASPEDASGFGLVSRRTSTSSASPIWNNTGSPPAQQTKLHIDEVTDQMQTFTPFVPYERYYDYGADMGAAGTGVGAGLPNVAGVAPSGLPASGAPIGPASMTMGGMPSMGGMPGMAGLTQSPQAPTAAVSNGHDFYGFRGRPSPRKYGVKEFFPNGLDDEVEVSKKKGSPEQIVAKPVTTRTPTNKASVVAAPEADIASVPATTAVPTTPTPPRVESRDTNSAASAPPSSSSRRKRDGYRSPLLEEFRNNKSKKFELKDLQGHIVEFSGDQHGSRFIQQQLESASGEEKSAIFEEIRPSSLQLMTDVFGNYVVQKFFVHGSNAQKAVLTKQMEGHVLSLSLQMYGCRVVQKAIEYVDTAKQAHLINELDKHVLRCVKDQNGNHVIQKAIEKIPPQHIQFIINAFNEQVYQLATHPYGCRVIQRMLEHCEEAQAAILAELHNYAYHLIQDQYGNYVIQHVLEQGAPDDKEAMMLVIKQHVLIFSRHKFASNVVEKCVIYGNRRQRRALIEEIATEREDGTLPITVMMKDQFANYVIQKLLDVSEGEDFDLLVSIIKPHLASLKKYSYGKHLASIERLVLLSEGGE

>Smik_smik_1210-g1.1

MDMDMDMELASIVSSLSALSNGNNNGSQATAIVGSATAGSQQIGGFRRSSFTTSNDIDSDILLLHGSSESSPIFKKTALSVGTAPPFSTNSKKFFGNGSNYYHYRSNDTSSLSSASHNNYQTHNAAGNLGKNNKVNHLLGQYSASIAGPVYYNGNENNNSGGEGFFEKFGKSLIDGTRELESQDRPEVTANQSQFIPESVSNASLDTQSTFEQTVQSDANSNNLNRNTTNSGSLYHSSSNSGSSASLESENAHYPKRNIWNVANTPVFRPSNSPAGVGASNVLVPNQQDGLANNNFPSYINGFPPNQFHQGPQYQNFPNYLIGSPSNFISQMISVQIPTNEDTENAGGKKKKKANRPSSVSSPSSPPNNTPFSFPYPNPMMFMPPPTPSATQQQHQQQQQQDQPQQQENPYIYYPSPTHIPVKVHTKEEKNFKKRNNKNHPANNTNNTNKQANPYLENLSTPKSSSKKNASSKSSESTGNSHKSQSQSQSQSQPQSSPHQQQQQMYHRSPLLEQLRSSGSDKNSNSSMSLKDIFGHSLEFCKDQHGSRFIQRELATSPASEKEVIFNEIRDDAIELSNDVFGNYVIQKFFEFGSKIQKDALVEQFKGHMKQLSLQMYACRVIQKALEFIDSKQRIELVLELSDSVLQMIKDQNGNHVIQKAIETIPIEKLPFILSSLTGHIYHLSTHSYGCRVIQRLLEFGSSNDQCSILNELKDFIPYLIQDQYGNYVIQYILQQNQFTNKEMVDVKQEIIETVANNVVEYSKHKFASNVVEKSILYGSKDQKDLIMSKILPKDKNHALNLEDDSPMILMIKDQFANYVIQKLVNVSEGEGKKLIVIAIRAYLDKLNKSNSLGNRHLASVEKLAALVENAEV
